# Strongly Bound Charge-Transfer Interface Excitons in Lateral Monolayer MoSe_2_–WSe_2_ Heterostructures

**DOI:** 10.1021/acs.nanolett.5c06500

**Published:** 2026-07-17

**Authors:** Vishal Suri, Sakal Singla, Suman Kumar Chakraborty, Vivekanand Jakhar, Suman Sarkar, Prasana Kumar Sahoo, Biswanath Chakraborty, Sabine Körbel

**Affiliations:** † Department of Physics, 528895 Indian Institute of Technology (IIT) Jammu, 181221 Jammu, India; ‡ Materials Science Centre, 30133 Indian Institute of Technology (IIT) Kharagpur, 721302 Kharagpur, India; ¶ Department of Materials and Metallurgical Engineering, 528895 Indian Institute of Technology (IIT) Jammu, 181221 Jammu, India; § Institute of Condensed Matter Theory and Optics, Friedrich Schiller University Jena, Max-Wien-Platz 1, 07743 Jena, Germany

**Keywords:** two-dimensional material, monolayer transition-metal dichalcogenide, lateral heterostructure, interface exciton, charge transfer, chemical vapor deposition, low temperature, photoluminescence spectroscopy, first-principles calculations, ab initio many-body perturbation theory, GW approximation, Bethe−Salpeter equation

## Abstract

In lateral heterostructures of two different transition-metal dichalcogenide monolayers with type-II band alignment, appropriately engineered interface widths and band offsets give rise to dipolar charge-transfer interface excitons, characterized by electrons and holes localized on opposite sides of the junction. This topic has been little explored, mainly because of difficulties to synthesize clean and uniform lateral interfaces. Here, using different experimental techniques, we systematically probe excitons in lateral monolayer MoSe_2_–WSe_2_ heterostructures with both sharp and diffuse junctions. Using low-temperature photoluminescence spectroscopy, we find an additional spectral signal exclusively at sharp junctions, compatible with a dipolar charge-transfer exciton. Parameter-free ab initio many-body perturbation theory yields stable, strongly bound dipolar charge-transfer excitons at the lateral MoSe_2_–WSe_2_ interface already for small lateral band offsets, supporting the assignment of our detected photoluminescence signal at sharp junctions to a dipolar charge-transfer interface exciton.

## Graphical abstract

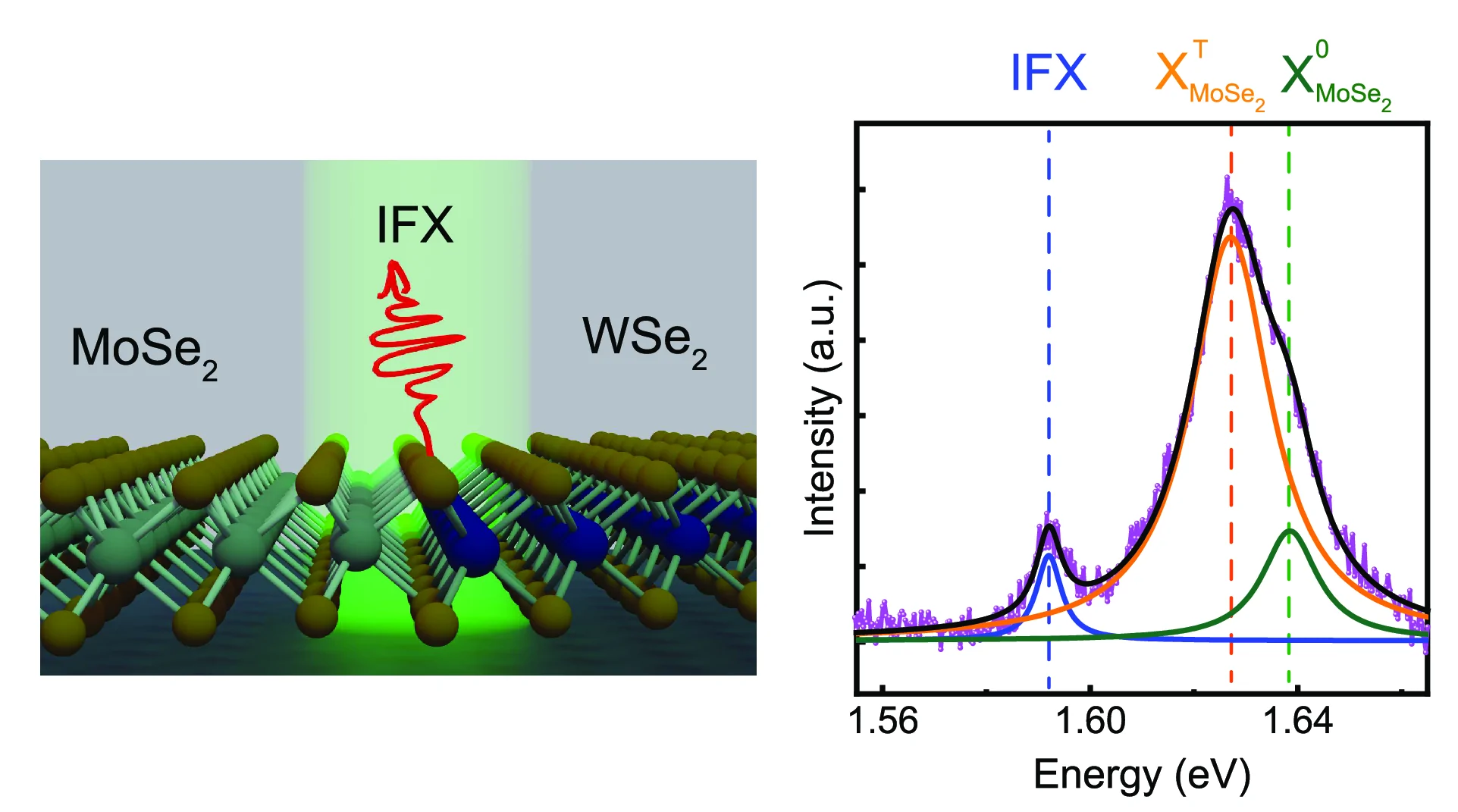

Vertical heterostructures (VH)^1^ of two different semiconducting two-dimensional (2D) transition-metal dichalcogenides (TMDs) with a type-II band offset can host interlayer excitons (ILX) with the electron localized on one layer and the hole on the other,^2^ like atomically narrow p–n junctions. Interlayer excitons can have long recombination lifetimes, possibly form condensates,^3,4^ and allow manipulation with electric fields due to their dipolar nature.^5^ Interlayer excitons^6^ in vertical heterostructures of monolayer TMDs, namely, MoSe_2_ and WSe_2_, have been detected with photoluminescence (PL) spectroscopy^7–11^ and investigated theoretically with first-principles many-body perturbation theory.^12–15^ Interlayer and intralayer excitons have similar binding energies of about 0.2 eV, the type-II band offset of 0.3–0.4 eV between MoSe_2_ and WSe_2_ in the vertical heterostructure renders the transition energy of interlayer excitons 0.2–0.4 eV below that of the A exciton in MoSe_2_.^15,16^ Lateral heterostructures (LH), where the two monolayer materials are attached in-plane at their edges (Figure 1a), have also been successfully synthesized using chemical vapor deposition (CVD) or epitaxy from different material combinations: hexagonal boron nitride (hBN)/graphene,^17–21^ MoS_2_/MoSe_2_ or WS_2_/WSe_2_,^22,23^ NbS_2_/WS_2_,^24^ TaSe_2_/MoSe_2_,^25^ MoSe_2_/WS_2_,^26^ and MoSe_2_/WSe_2_ or MoS_2_/WS_2_.^27–47^ The formation of sharp and diffuse interfaces in lateral MoSe_2_–WSe_2_ heterostructures was ascribed to the energetics of atomic adsorption on the TMD edge, and different type-II band offsets up to 0.3 eV were observed at different lateral interfaces using scanning tunneling spectroscopy.^36^ Synthesis, properties, and applications of 2D lateral heterostructures are reviewed in, e.g., refs 48–53 , theoretical investigations are compiled in ref 54.

**Figure 1:**
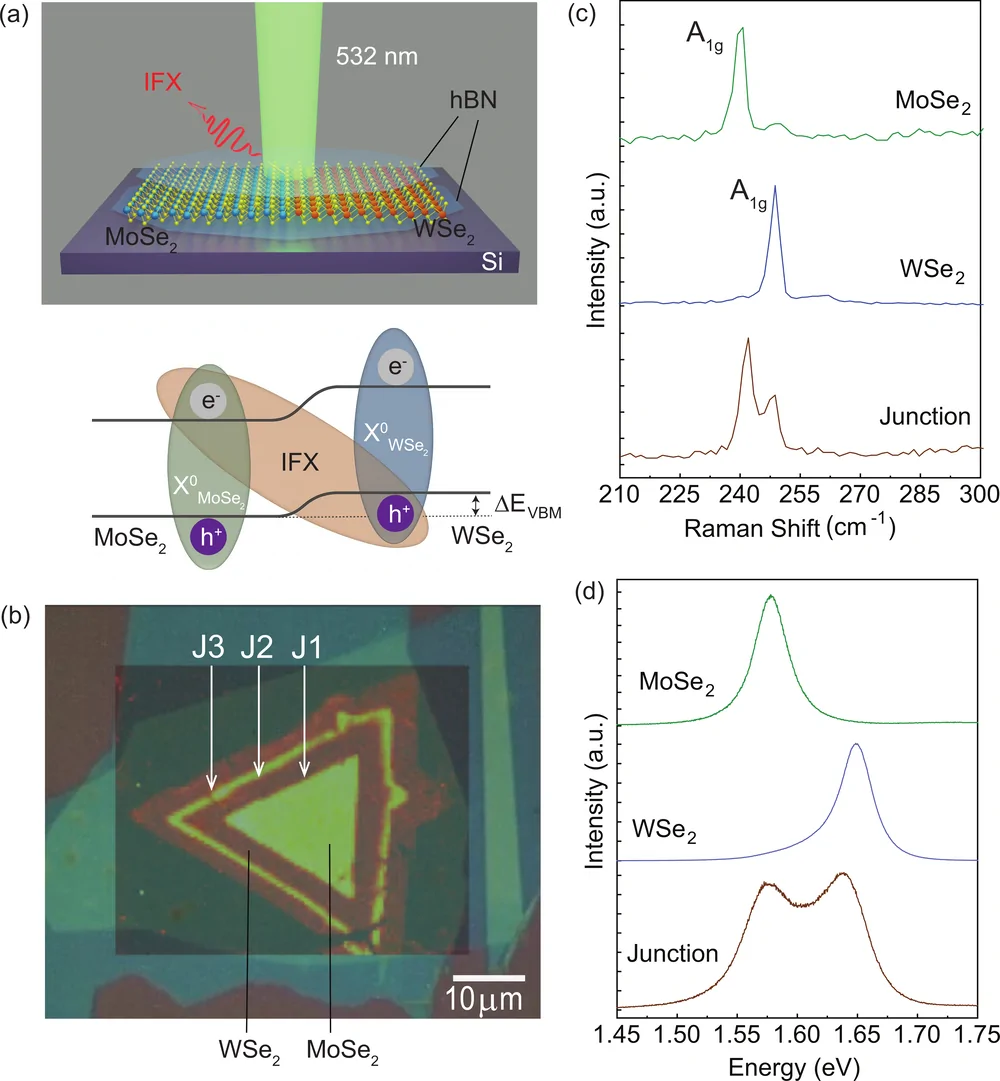
Schematic and room temperature measurements. (a) Schematic of the IFX emission at the lateral heterojunction of MoSe_2_ and WSe_2_. Δ*E*_VBM_ is the valence-band offset. (b) Raman map overlaid with the optical image of the hBN encapsulated MoSe_2_–WSe_2_ lateral heterostructure. Yellow and red regions correspond to MoSe_2_ and WSe_2_ respectively. (c and d) Raman and PL spectra from MoSe_2_, WSe_2_, and their junction, respectively, at room temperature.

Recently, a few studies suggested the existence of dipolar charge-transfer (CT) interface excitons (IFX) in lateral heterostructures. Such charge-transfer interface excitons confined to one-dimensional lateral interfaces can reach high densities^47^ that may enable observing unusual phenomena like exciton condensation. Dipolar charge-transfer interface excitons with the hole located on one side of the interface and the electron on the other have a permanent dipole moment allowing manipulation with electric fields, with potential applications in excitonic circuitry.^55^ Whether a dipolar charge-transfer exciton can form at type-II interfaces (Figure 1a) depends on the trade-off between the band offset (large is favorable) and strong electron–hole attraction (not favorable). Sharp interfaces are more likely than broad ones to host dipolar charge-transfer interface excitons since they allow smaller electron–hole distances. Yuan et al.^56^ reported a PL signal in a lateral WSe_2_–W­(S,Se)_2_ heterostructure about 0.3 eV below that of the A exciton in WSe_2_ that was attributed to a dipolar charge-transfer interface exciton. Lau et al.^57^ investigated interface excitons in free-standing, atomically sharp MoSe_2_–WSe_2_ heterojunctions using a real-space tight-binding approach and a perturbation expansion with a hydrogen-like basis in the effective-mass approximation. They investigated excitonic binding energy, exciton radius, and optical dipole matrix element of interfacial charge-transfer excitons as a function of the type-II band offset, and obtained exciton binding energies of about 0.2 eV (to be compared to about 0.3 eV for intradomain excitons), an exciton radius between 1 and 5 Å, and a reduction in optical dipole matrix element up to several orders of magnitude. For a band offset of 0.1 eV, corresponding to the MoSe_2_–WSe_2_ LH, Lau et al. found 0.25 eV excitonic binding energy, an exciton radius of 1.7 nm, and a reduction in dipole matrix element by only 10%. In lateral MoSe_2_–WSe_2_ heterostructures,^44^ a PL signal observed only at the interface about 80–100 meV below the A exciton in MoSe_2_ was also attributed to a dipolar charge-transfer interface exciton. In ref 44, Rosati et al. investigated exciton binding energy, energetic distance from the A exciton in MoSe_2_, and electron–hole distance of the interfacial charge-transfer exciton as a function of type-II band offset, interface width, and dielectric screening using an effective-mass model. They found exciton binding energies of about 200 meV for CT excitons in free-standing LH, similar to Lau et al., but only 30–40 meV in LH encapsulated by hexagonal boron nitride, hence a band offset of 200 meV would be needed to form dipolar charge-transfer interface excitons. In their simulation, in agreement with their experiment, the CT exciton in the encapsulated LH with 2–3 nm width was observed about 88 meV below the A exciton in MoSe_2_.

Parameter-free first-principles methods, such as the GW approximation and the Bethe–Salpeter equation (BSE), that account for the strong electron–hole attraction constitute a complementary approach in addition to model Hamiltonians. Such a study has been missing, a gap that we fill in this work. Previous first-principles studies of lateral monolayer heterostructures investigated different properties (electronic structure,^56,58–61^ band alignment,^62–65^ strain,^65–69^ phonons,^70,71^ defects,^72^ molecule adsorption,^73,74^ electronic transport,^75–82^ spin–orbit coupling,^83^ interaction with plasmons,^84^ and interface reconstruction^85^) for different material combinations [MoSSe/WSSe,^60,61^ MoS_2_/MX_2_ (M = Mo, W; X = S, Se),^86,87^ MX (M = Ge, Sn, Ga, In; X = S, Se, Te),^88,89^ MoTe_2_/WTe_2_ ^90,91^ ], but only a few works explicitly considered interface excitons. One of them did not consider the important electron–hole interaction.^92^ In others, ab initio molecular dynamics combined with real-time time-dependent density functional theory was employed to investigate electronic excitations in lateral MoS_2_–WSe_2_,^64^ MoS_2_–WS_2_,^93^ MoSe_2_–WSe_2_,^71^ and W­(S,Se)_2_–Mo­(S,Se)_2_ heterostructures.^94^ An interface exciton was observed with the hole located on the WSe_2_ side and the electron’s density centered at the MoS_2_–WSe_2_ interface.^64^ This approach takes electron–phonon interaction into account, however, it is not clear if and how electron–hole interaction was considered, and these studies did not investigate interface excitons in detail.

In this work, we investigate lateral MoSe_2_–WSe_2_ heterostructures featuring both sharp and diffuse interfaces. Low-temperature (*T* ∼ 5 K) PL measurements uncover an additional peak that emerges exclusively at the sharp lateral junctions and is absent in their diffuse counterparts. We designate this feature as the IFX. To elucidate its origin, we perform parameter-free first-principles calculations within many-body perturbation theory,^95^ combining the GW approximation for accurate quasiparticle band structures with the BSE for excitonic states. This theoretical approach has strong predictive power, as it is directly based on Schrödinger’s equation for electrons and ions, requires no prior assumptions about the electronic structure at the interface, and takes into account the strong electron–hole interaction in 2D TMD.^96–98^ Our approach yields besides the oscillator strength also the polarization direction of the excitonic emission, both of which are determined by transition dipole moments.

The upper panel of Figure 1a schematically depicts PL emission by the interface excitons formed in the lateral heterostructure. The lateral heterostructure exhibits type-II band alignment, as shown in the lower panel of Figure 1a. This alignment facilitates the formation of dipolar charge-transfer interface excitons, composed of an electron and a hole that reside on opposite sides of the interface.

Following earlier reports,^99^ a one-pot water-assisted CVD strategy is used to synthesize the MoSe_2_–WSe_2_ LH, which allows us to precisely control the thickness and covalently bonds the two phases laterally. Figure S1a shows the optical image of the fabricated LH grown on silicon wafer with a 285 nm thick oxide layer as the substrate. Furthermore, the fabricated LH is encapsulated by thin hBN flakes using a dry transfer technique.^100^ We performed steady-state photoluminescence spectroscopy and Raman spectroscopy under 532 nm laser excitation at room temperature on the LH samples. Figure 1b shows the optical image overlaid with the Raman map of the hBN-encapsulated MoSe_2_–WSe_2_ LH (sample LH1). The Raman map consists of two regions: Yellow (MoSe_2_) and red (WSe_2_) with the Raman shift centered at ∼240 and ∼248 cm^–1^, respectively. We observed A exciton emission peaks from MoSe_2_ and WSe_2_ at ∼1.57 eV (790 nm) and ∼1.65 eV (751 nm), respectively (Figure 1d). Overall, the monolayer characteristics of MoSe_2_ and WSe_2_ have been confirmed by optical microscopy, Raman spectroscopy, and PL spectroscopy.

Earlier studies have shown that interlayer excitons (ILXs) in vertical heterostructures of TMDs can be observed at room temperature,^101^ but their intensity is very weak because the thermal energy overcomes the binding energy of ILXs, causing them to dissociate into free electrons and holes. This causes a reduction in the number of ILXs available for radiative recombination. With decreasing temperature, the ILX peak intensifies and dominates the PL spectra at extremely low temperatures (∼5 K).^11^ We performed room-temperature steady-state PL spectroscopy at different regions of our encapsulated lateral heterostructures (samples LH1 and LH2) and observed the excitonic features of MoSe_2_ and WSe_2_ at the junction. However, we did not observe any IFX features unlike the case of ILX in vertical heterostructures. This could be due to the following two reasons. First, for the same laser excitation spot, vertical heterostructures support a much larger population of interlayer excitons since the junction extends over a plane, whereas in lateral heterostructures the interface excitons are confined to a narrow one-dimensional junction line. Second, the binding energy of IFXs in lateral heterostructures is expected to be different from that of ILXs in vertical heterostructures made of the same two materials because of the different bonding nature–van der Waals bonding in vertical heterostructures and covalent bonding in lateral heterostructures. For instance, the energy separation between interlayer exciton and the MoSe_2_ trion peak in vertical MoSe_2_–WSe_2_ heterostructures is reported to be ∼220 meV,^101^ whereas that for lateral heterostructures is reported to be ∼50 meV.^44^ The PL spectra from the junction regions for samples LH1 and LH2 are shown in Figures 1d and S2d, respectively.

The hBN-encapsulated MoSe_2_–WSe_2_ LH samples (LH1, LH2, and LH3) were cooled to 5 K using a Montana cryostat with a high vacuum (10^–5^ Torr). PL measurements were taken by illuminating the samples with a 532 nm CW laser. Figure 2a shows the main experimental findings of our present investigation. The contour map (Figure 2a) is generated from a line scan taken along a direction nearly perpendicular to the three junctions J1, J2, and J3 of sample LH1. The line scan is taken with a step size of 0.2 μm and a laser power of 3 μW for an integration time of 10 s at each step. The inset shows the optical image superimposed on the PL map of the LH, where the three junctions are marked as J1, J2 and J3. The line scan is initiated within the MoSe_2_ domain and progresses toward the WSe_2_ region, sequentially intersecting the three lateral junctions (J1, J2, and J3) along its measurement path. At the junction J2 a weak peak at ∼1.59 eV appears in addition to the MoSe_2_ (1.63 eV) and WSe_2_ (1.72 eV) peaks, marked by a white rectangle in Figure 2a, with the corresponding spectrum shown in Figure 2b. We attribute the 1.59 eV feature to the PL emission peak of the IFX, as it appears specifically at the interface. At junction J3, we observe the peaks of MoSe_2_, WSe_2_, and a very weak signal of the IFX. A similar trend of the PL emission is observed in samples LH2 and LH3 (Figures S3 and S4, respectively). High-angle annular dark-field (HAADF) imaging reveals that J2 possesses an atomically sharp interface, J1 exhibits a relatively diffuse interface, while J3 shows an intermediate interfacial sharpness, consistent with our earlier report^99^ (Figure S11). According to ref 36, sharp interfaces tend to form when MoSe_2_ is grown on WSe_2_ (as in the case of our junction J2), whereas diffuse interfaces form when WSe_2_ is grown on MoSe_2_ (as in the case of our junctions J1 and J3). In ref 36, this observation is ascribed to different energetics of atomic adsorption at the interfaces depending on the order of deposition. At broad lateral interfaces the charge-transfer exciton should have a larger exciton radius and/or experience a smaller effective band offset, reducing its stability and bringing it closer in energy to the A exciton in MoSe_2_ where it is harder to detect, in line with the prediction of Rosati et al.^44^ A slight Raman peak shift is observed for both MoSe_2_ and WSe_2_ at the lateral interface (Figures S12 and S13), consistent with weak local strain that naturally arises during lateral heterostructure formation. However, the magnitude of this strain remains small and is not expected to significantly alter the interfacial band alignment.

**Figure 2:**
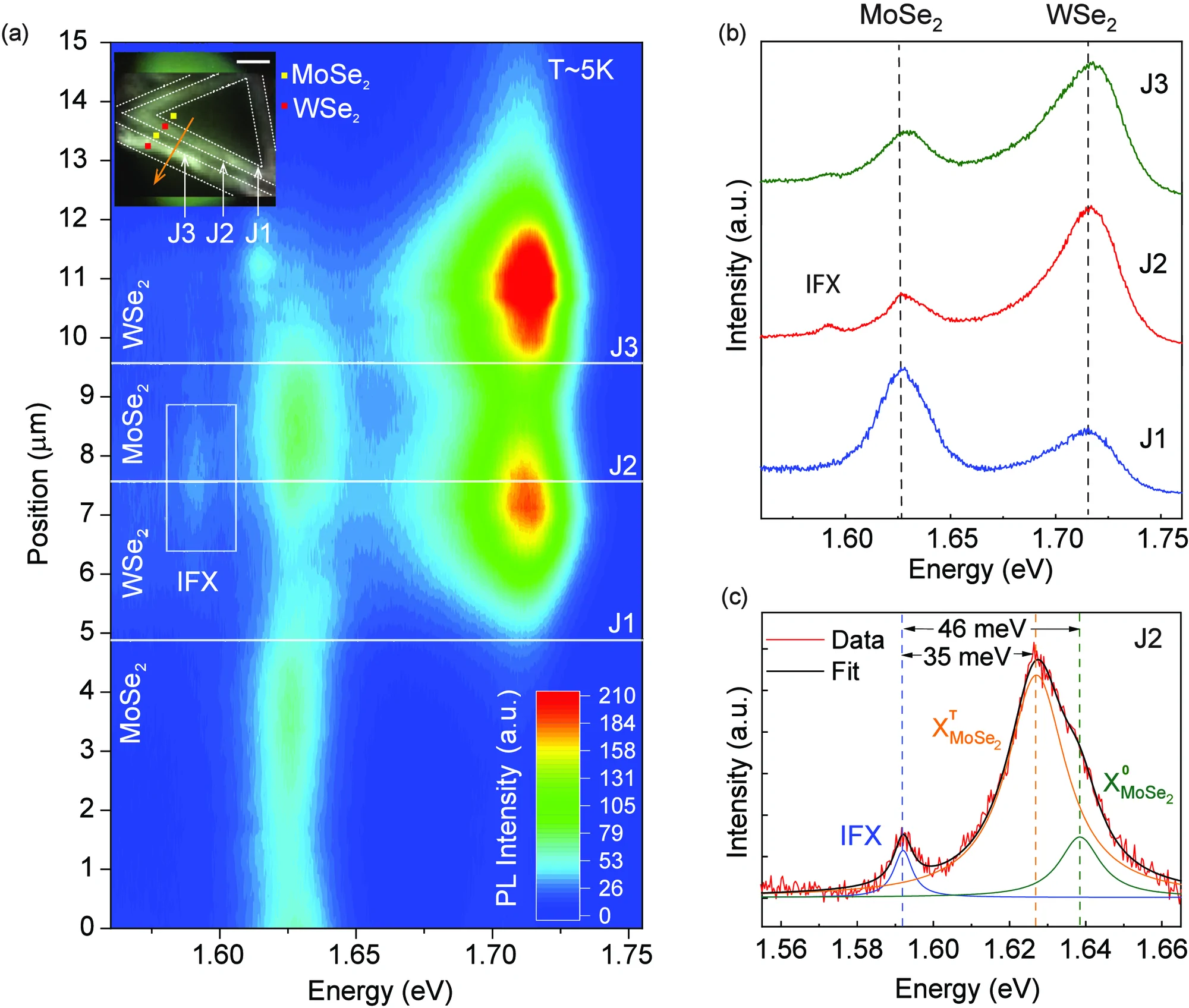
Low-temperature measurements. (a) Contour map of PL from the hBN-encapsulated lateral MoSe_2_–WSe_2_ heterostructure (sample LH1) at *T* ∼ 5 K, excited with a 532 nm CW laser at a power of ∼3 μW. Apart from the low-temperature excitonic peaks of MoSe_2_ (1.63 eV) and WSe_2_ (1.72 eV), an additional peak (1.59 eV) is observed at the sharp junction J2 only, marked by the white rectangle. This peak is absent at the smooth junctions J1 and J3. Inset: Optical image of hBN-encapsulated LH superimposed on the PL map at 5 K. Scale bar: 5 μm. The scan was taken along the line marked by the orange arrow. (b) PL spectra from the three junctions at 5 K. (c) PL spectrum from junction J2 fitted with Lorentzian functions. The interface exciton, the MoSe_2_ A exciton, and the MoSe_2_ trion are marked as IFX, $X_{MoSe_{2}}^{0}$ , and $X_{MoSe_{2}}^{T}$ , respectively. The IFX peak energy lies 35 meV below the trion and 46 meV below the exciton peak of MoSe_2_.

Figure 2c shows the Lorentzian fitting of the ∼1.63 eV peak of MoSe_2_ from sample LH1, consisting of the trion $\left(X_{MoSe_{2}}^{T}\right)$ and A exciton $\left(X_{MoSe_{2}}^{0}\right)$ components at ∼1.627 and ∼1.638 eV, respectively. The IFX peak is centered at ∼1.592 eV with a line width of ∼6 meV. The corresponding fitting for LH2 and LH3 is shown in Figures S3c and S4c, respectively. For LH2, the IFX peak is centered at ∼1.583 eV with a line width of ∼15.9 meV, while LH3 exhibits an IFX peak centered at ∼1.583 eV with a line width of ∼4.9 meV.

We performed a temperature-dependent PL study of the IFX peak observed at the sharp junction. Figure 3a shows the evolution of the IFX with temperature, where it is fitted with Lorentzian functions. We observe that the PL intensity of the IFX decreases with increasing temperature and vanishes at *T* ∼ 45 K. Figure 3b shows the temperature dependence of the PL intensity extracted from the Lorentzian fitting from Figure 3a. We observe that the integrated intensity decreases monotonically with increasing temperature. This may originate in thermally driven carrier delocalization and enhanced nonradiative recombination which suppress radiative emission,^102^ or in escape of the IFX from the interface by thermally assisted transformation into, e.g., the A exciton in MoSe_2_.^40,103^ Since the IFX has a large binding energy of several hundred millielectronvolts (see below), we do not expect that charge-carrier separation is responsible. The blue-shift with decreasing temperature may be caused by stronger dipole–dipole repulsion at higher exciton densities. We fit the integrated PL intensity with a power law

1 $$I=AP^{\alpha }$$

­(*I* and *P* are integrated PL intensity and incident laser power, respectively) and find an inverse dependence of the integrated intensity on temperature (α = −1.066). Figure 3c shows the excitation power dependence of the integrated intensity between 3 and 60 μW. We fit the integrated intensity again with a power law (eq 1) and find almost a linear integrated intensity dependence on excitation power (α = 0.9754). The integrated PL intensity exhibits a linear dependence on excitation power because the emitting states are not constrained by a finite density of states. In contrast to defect-related emission, which saturates and produces a sublinear response once available traps are filled,^104^ these states scale proportionally with the photogenerated exciton population, thereby maintaining linear growth. The variation of peak position of IFX with temperature and power is shown in parts a and b of Figure S7, respectively. The corresponding experimental PL spectra used to extract the integrated PL intensity are given in Figure S5. The lateral interface breaks in-plane rotational symmetry^57^ and enables exciton emission with a preferential in-plane polarization direction, other than homogeneous MoSe_2_ or WSe_2_, whose in-plane component of emission should be isotropic.^105^ We performed polarization-dependent PL spectroscopy at the sharp junction J2, where the interface exciton was observed. The polar plot in Figure 3d shows the PL intensity of the IFX as a function of polarizer angle in the monolayer plane measured at 10° intervals from 0° to 180°, where 0° corresponds to the laser polarization. Each spectrum is taken by rotating the polarizer in the output path. The details of the measurement setup are discussed in Section 8 and Figure S8a. The black circles are the experimental data, which is fitted by the function (solid red line) given by

2 $$I=I_{0}\cos^{2}\left(\theta -\theta_{0}\right)$$

with *I*_0_ = 20.12 and θ_0_ ≈ 50°. Indeed we observe polarized exciton emission for the IFX with the in-plane polarization perpendicular to the interface. This polarization angle does not align with that of the laser. We observed that the IFX emission is polarized at 50° with respect to the laser polarization. Such a well-defined polarization response is indicative of excitons possessing a fixed transition dipole moment,^106^ which in our case is oriented perpendicular to the interface. The integrated PL intensity as a function of polarizer angle, plotted in Cartesian coordinates, is presented in Figure S7c, and the corresponding experimental PL spectra used to extract the integrated PL intensity are given in Figure S5. The polarization direction of the emission is given by that of the electronic transition moment $d_{t}∼e\left\langle \psi_{v}\left|r\right|\psi_{c}\right\rangle$ between valence and conduction states *v* and *c*, which couples to an electric field **E** in the form $f∼|d_{t}\cdot E{|}^{2}$ , where *f* is the oscillator strength.^107^ Our observed polarization direction indicates that the IFX couples to electric fields directed across the interface, as expected for an electronic transition with charge transfer across the interface. The variation of the integrated PL intensity as a function of temperature, excitation laser power, and polarizer angle for sample LH3 is shown in Figure S9. Excitation polarization-dependent measurements were performed on sample LH3. The corresponding polarization-resolved results are provided in Figure S10, demonstrating that the IFX emission remains unchanged upon varying the incident laser polarization. The polarization of the incident laser was varied using a half-wave plate (HWP) in the input path.

**Figure 3:**
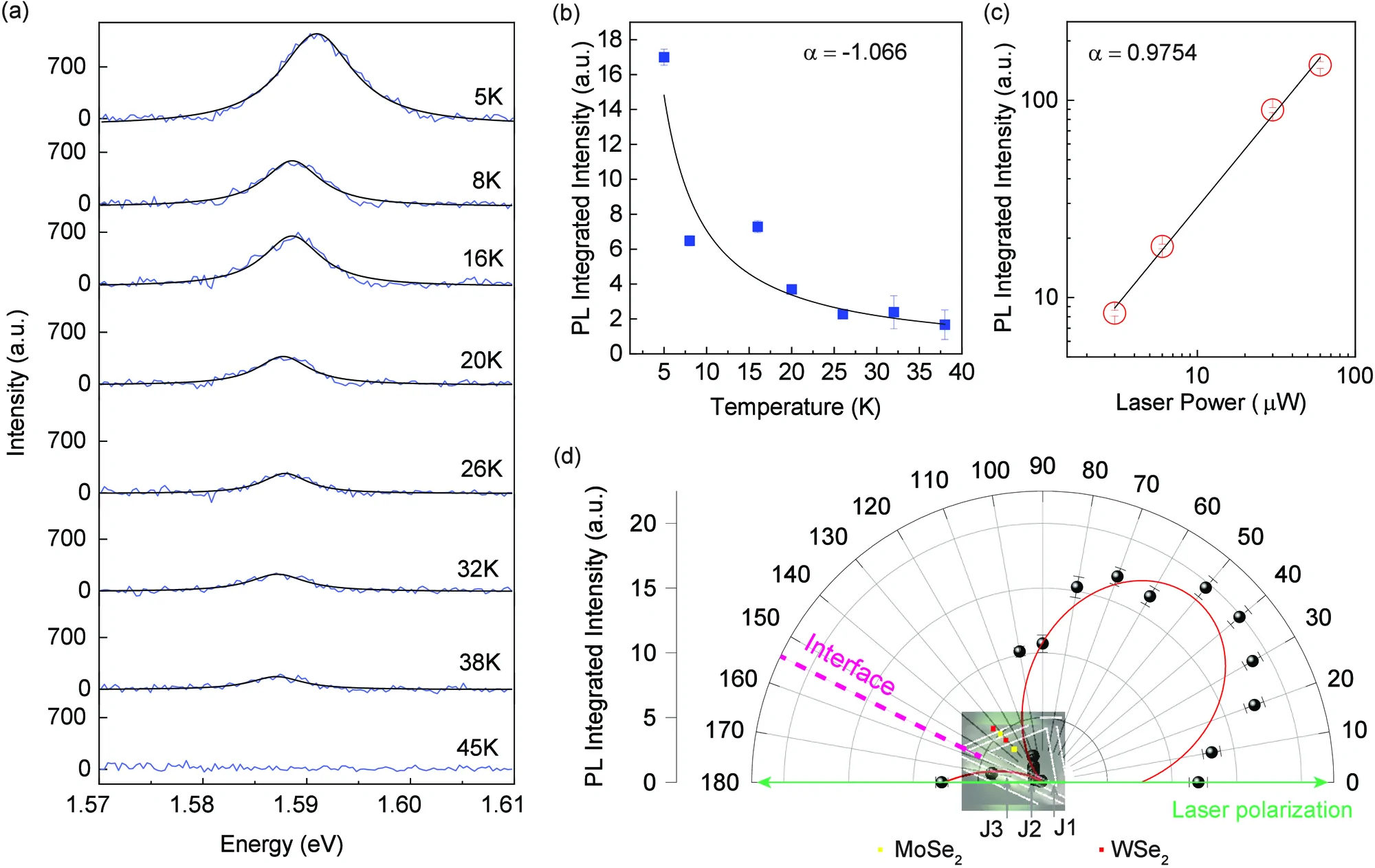
Temperature, power, and polarization dependence of the IFX. (a) Variation of the IFX’s PL spectrum with temperature. With decreasing temperature, the IFX blue-shifts and gains intensity. (b) Integrated PL intensity as a function of the temperature. A power law with an exponent of −1.066 gives almost an inverse PL intensity dependence on temperature. (c) Integrated PL intensity as a function of the excitation power. A power law with an exponent of 0.9754 gives almost a linear PL intensity dependence on excitation power between 3 and 60 μW. (d) Polar plot for integrated PL intensity obtained from the Lorentzian fits of the IFX measured at 10° intervals from 0° to 180°. The black circles represent the experimental data, while the red solid line depicts the fitted function. The double arrowed solid green line marks the laser polarization, whereas the magenta dashed line marks the interface. PL map overlaid with polar plot indicates that the interface is nearly perpendicular to the direction of maximum PL intensity, implying a transition dipole normal to the interface. Scale bar: 5 μm.

To gain additional insight into interface excitons in lateral heterostructures and the nature of the here observed IFX, we employed first-principles many-body perturbation theory. The electronic band structure was modeled using the *GW* approximation, starting from density functional theory in the generalized-gradient approximation. Excitonic states (eigenvalues, oscillator strengths, binding energies, wave functions) were determined by solving the BSE using the Vienna Ab initio Software Package (VASP)^109^ [see the Supporting Information (SI) and refs 110–113 for details]. The first-principles calculations were performed for a periodic array of lateral interfaces (Figure 4a). To limit the large computational cost of the simulations, we set the lateral extension of each stripe to about 35 Å, considerably smaller than in our experiment (≳1 μm) but comparable to other experiments^114^ and to the lateral extension of the excitonic wave function in vertical heterostructures.^12^ The spatial confinement of the excitonic wave functions should lead to a blue-shift and to quantization in the dimension of confinement and should pose an upper limit of ≈3.5 *e*·nm to the excitonic dipole moment. To allow comparison with our experiment, we red-shift the modeled excitonic spectrum of the heterostructure *a posteriori* by 0.09 eV (the energy difference between the brightest excitons in MoSe_2_ and the heterostructure). All excitons in the simulation are close to an interface and affected by it, and are in this sense interface excitons. We classify the computed interface excitons further based on their different spatial distribution of electron and hole (see the Methods section in the SI for details): IFX mainly localized in MoSe_2_ ${[\mathrm{“IFX}}_{MoSe_{2}}$ ”, green in Figure 4b–e], in WSe_2_ ${[\mathrm{“IFX}}_{WSe_{2}}$ ”, blue in Figure 4b–e], and dipolar charge-transfer interface excitons [“IFX_CT_”, red in Figure 4b–e]. The spatial distribution of electron and hole of the lowest-energy IFX are depicted in Figure 4b. We examined the lowest 120 excitonic states in detail. All but three exhibit charge transfer and a permanent dipole moment [eq. (4) in the SI] of ≈1–2 *e*·nm directed across the interface (Figure 4c). The dipole moments hence remain below the upper limit of ≈3.5 *e*·nm imposed by the width of the lateral stripes in the simulation, indicating that the dimensions of the simulation box are sufficient to give an indication of the spatial extension of the low-lying charge-transfer excitons. The interface excitons ${\mathrm{“IFX}}_{MoSe_{2}}$ ” and “ ${\mathrm{IFX}}_{WSe_{2}}$ ” with their smaller charge-transfer component have smaller dipole moments than “IFX_CT_”. When modeling photoluminescence we assume a broad energy distribution of the excitons (PL ∼ absorption). Our steady-state PL experiment should lie in-between this limit and that of thermalized excitons. IFX_CT_^(1)^ is one of the lowest visible PL peaks, well separated from the next bright peak, ${\mathrm{IFX}}_{MoSe_{2}}^{\left(2\right)}$ , by 48 meV (Figure 4d), in excellent agreement with experiment (46 meV below the A exciton in MoSe_2_). Thus, the interface exciton in the lateral heterostructure lies closer in energy to the MoSe_2_ exciton than the interlayer exciton in vertical heterostructures, whose energy lies 0.2–0.4 eV below the bright MoSe_2_ A excitons.^15,16^ The energy of ${\mathrm{IFX}}_{MoSe_{2}}^{\left(2\right)}$ is close to that of ${\mathrm{IFX}}_{CT}^{\left(1\right)}$ , and its intensity is slightly weaker, so that it might not be detected as a separate peak in experiment. Its polarization direction along the interface is not compatible with the experimentally observed polarization of the IFX. IFX_CT_^(1)^ is mostly polarized out-of-plane, which may help to distinguish it from other interface excitons. The out-of-plane component of the polarization of ${\mathrm{IFX}}_{CT}^{\left(1\right)}$ may be related to charge transfer between transition-metal (inner) and chalcogenide (outer) layers of the TMD monolayer. Since the simulation (small distance between interfaces leading to quantum confinement) differs from the real system, and since there are many interface excitons, we cannot with certainty assign a computed IFX to the observed one. IFX_CT_^(1)^ is a likely candidate. Its polarization component in the monolayer plane is hard to assess in the calculation due to its low intensity, but we obtain a nonzero in-plane component across the lateral interface, compatible with the experimentally observed polarization direction. One of the brightest IFX ( ${\mathrm{“IFX}}_{CT}^{\left(8\right)}$ ”) exhibits strong charge transfer (Figure 4b, top panel), showing that dipolar interface excitons can be bright in spite of spatial distance between electron and hole. The lowest computed visible optical in-plane transition at the interface is an ${\mathrm{IFX}}_{WSe_{2}}$ mixed with a small amount of IFX_CT_ (Figure 4b, bottom panel). We assign this peak to the dark A exciton in WSe_2_ ^115^ becoming weakly visible by coupling to an IFX_CT_. The energy of this exciton is possibly predicted too low due to underestimation of the energy of the WSe_2_ exciton relative to the MoSe_2_ exciton, similar to previous computational studies of TMD monolayers using a similar approach.^15,116–119^

**Figure 4:**
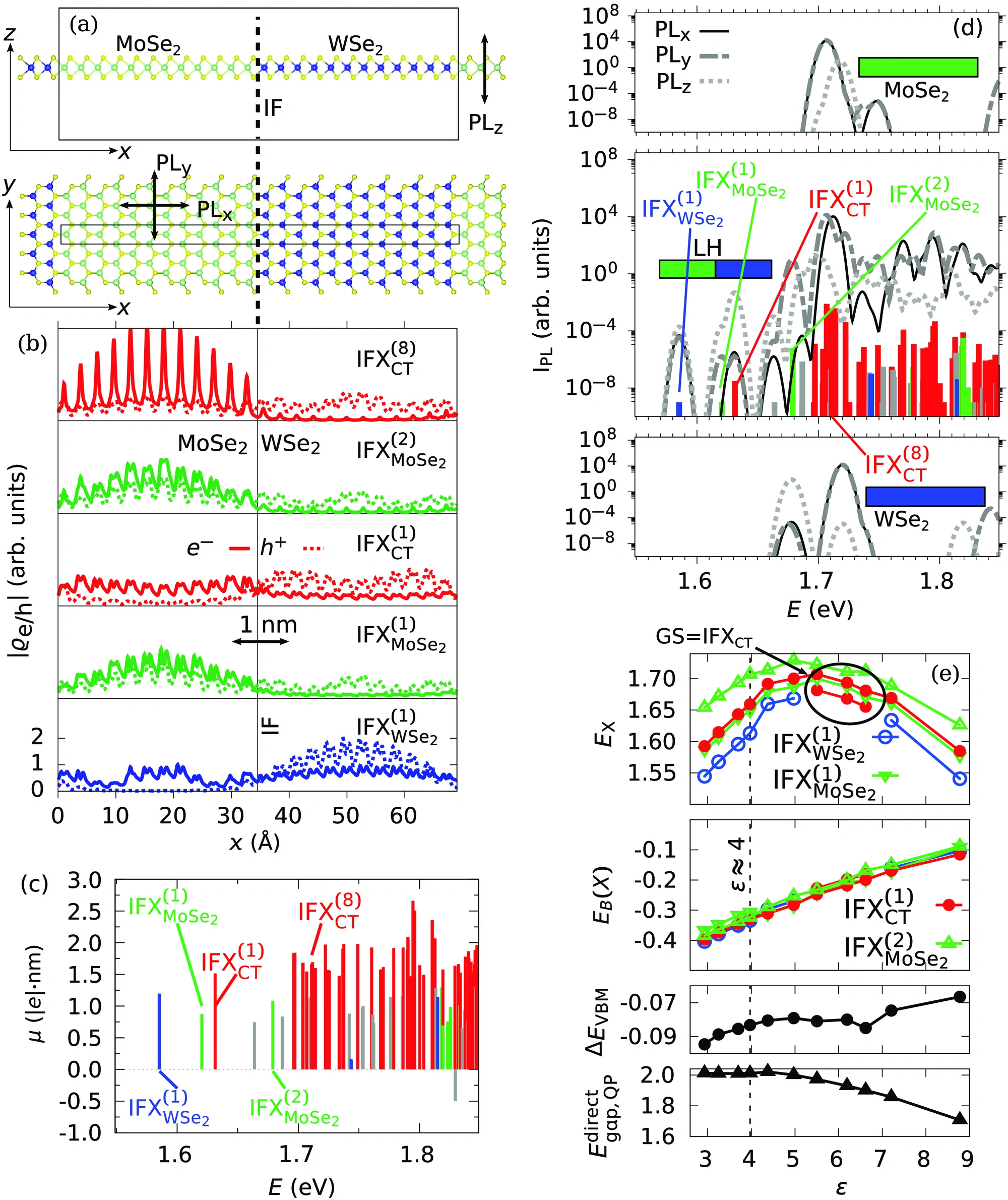
(a) Simulation box, (b) approximate density of electron (solid lines) and hole (dashed lines) for the lowest IFXs, (c) exciton dipole moment μ, (d) PL intensity spectra I_PL_ of MoSe_2_, the lateral heterostructure (LH), and WSe_2_ for the polarization directions PL_*x*_, PL_*y*_, and PL_*z*_ depicted in part a. Bars show the direction-averaged intensity of the individual excitonic transitions. (e) Exciton energy *E*_X_ and binding energy *E*_B_(*X*) for the lowest IFX (GS: ground state) and valence-band offset Δ*E*_VBM_ and direct electronic gap $E_{gap,QP}^{direct}$ (all in eV) of the LH as a function of the relative electronic dielectric permittivity ε. In parts b–d, ε ≈ 4, close to that of hexagonal boron nitride.^108^

The influence of dielectric screening ε on the energies of the lowest interface excitons is nonmonotonous (Figure 4e, upper panel). The interface excitons’ emission energies are the sum of the nonlinearly ε-dependent direct quasiparticle gap $E_{gap,QP}^{direct}$ and the almost linearly ε-dependent exciton binding energy *E*_B_(*X*) (Figure 4e, center panel). Importantly, the binding energy of dipolar charge-transfer interface excitons is large (≈0.3 eV), very close to that of the A excitons in MoSe_2_ and WSe_2_, in spite of strong charge transfer across the interface, similar to interlayer excitons in vertical heterostructures.^15^ For 5 ≤ ε ≤ 7, the lowest-energy interface exciton is a dipolar charge-transfer exciton. The valence-band offset Δ*E*_VBM_ between MoSe_2_ and WSe_2_ is ≈0.1 eV and depends only weakly on ε. This small Δ*E*_VBM_ is in agreement with previous findings that lateral heterostructures exhibit smaller band offsets than vertical heterostructures.^63,64^

Similar to the findings of Lau et al. and Rosati et al. for the free-standing case, we find a large exciton binding energy of about 300 meV of the interfacial CT exciton also for the encapsulated case, and observe that a higher dielectric permittivity weakens the excitonic binding energy of both CT and MoSe_2_ excitons by a similar amount, so that dipolar charge-transfer interface excitons can form already at a small band offset of ≈0.1 eV. Our first-principles results largely agree with those from the model Hamiltonians of refs 57 and 44, except that we find a more complex excitonic spectrum and a larger binding energy of interfacial charge-transfer excitons, especially for encapsulated lateral heterostructures. Similar to Rosati et al.,^44^ we find the dipolar charge-transfer exciton to be the lowest-energy exciton state for intermediate ε (Figure 4e, top panel).

We cannot exclude gap states arising from atomic reconstruction at the lateral interface^85^ contributing to the interface exciton’s emission. Still, the calculation results show that strongly bound dipolar charge-transfer excitons dominate the low-energy range of the emission spectrum and that these low-energy excitons have a permanent dipole moment across the lateral interface.

Concluding, we have investigated excitons at lateral interfaces in monolayer MoSe_2_–WSe_2_ heterostructures, combining experiments and theoretical modeling. At temperatures below ≈45 K, a photoluminescence signal about 46 meV below the A exciton in MoSe_2_ is observed at the sharp interface that is absent in the diffuse/broader junctions. Our first-principles electronic-structure calculations based on many-body perturbation theory yield a dipolar charge-transfer exciton (dipole moment ≈1.5|*e*| nm that fits well to the observed interface width of 1 nm) with electron and hole on different sides of the interface and a transition energy 48 meV below the exciton in MoSe_2_, very similar to the experimentally observed exciton. This interface exciton has a strong binding energy (≈0.3 eV) close to that of the A excitons in MoSe_2_ and WSe_2_ in spite of strong charge transfer across the interface. Our experimental and theoretical evidence combined make a strong case for strongly bound dipolar charge-transfer excitons at lateral interfaces in monolayer MoSe_2_–WSe_2_ heterostructures.

## Supplementary Material



## Notes

Details of experimental and theoretical methods, optical images of nonencapsulated and hBN-encapsulated lateral MoSe_2_–WSe_2_ heterostructures, room temperature characterization of sample LH2, low-temperature results of samples LH2 and LH3, temperature, power, and polarization dependence of the PL signal of the interface exciton for LH1 and LH3 along with the measurement set up schematic, HAADF images of the junctions, excitation polarization-independent anisotropic IFX emission in sample LH3, Raman analysis of samples LH1 and LH3, convergence tests for the first-principles calculations, and calculation parameters (PDF)
