# Supplementary material for: Strongly Bound Charge-Transfer Interface Excitons in Lateral Monolayer MoSe2–WSe2 Heterostructures
Source: Nano Lett. 2026 Jul 17;26(35):11634–44. doi: 10.1021/acs.nanolett.5c06500 (PMC13564875; doi:10.1021/acs.nanolett.5c06500)
Supplement: Supplementary file 1 [file nl5c06500_si_001.pdf]

Supporting Information
Strongly bound charge transfer interface excitons in lateral monolayer $MoSe_2$-WSe_2 heterostructures

Vishal Suri,[†] Sakal Singla,[†] Suman Kumar Chakraborty,[‡] Vivekanand Jakhar,[¶] Suman Sarkar,[¶] Prasana Kumar Sahoo,[‡] Biswanath Chakraborty,[*,†] and Sabine Körbel[§]

†*Department of Physics, Indian Institute of Technology Jammu, 181221 Jammu, India*

‡*Materials Science Centre, Indian Institute of Technology Kharagpur, 721302 Kharagpur, India*

¶*Department of Materials and Metallurgical Engineering, Indian Institute of Technology Jammu, 181221 Jammu, India*

§*Institute of Condensed Matter Theory and Optics, Friedrich Schiller University Jena, Max-Wien-Platz 1, 07743 Jena, Germany*

E-mail: biswanath.chakraborty@iitjammu.ac.in

Contents

Methods. 3

Experimental methods. 3

First-principles Calculations. 4

Additional results from microscopy and spectroscopy. 7
Figure S1: Optical images of non-encapsulated and hBN encapsulated $MoSe_2$-WSe_2 lateral heterostructures. 7
Figure S2: Room temperature characterisation of sample LH2. 8
Figure S3: Low temperature results of sample LH2. 9
Figure S4: Low temperature results of sample LH3. 10
Figure S5: Temperature-dependent and Power-dependent data of LH1 sample. 11
Figure S6: Polarization-dependent data of LH1 sample. 12
Figure S7: Additional analysis of IFX observed in LH1 sample. 13
Figure S8: Polarization-resolved PL measurement geometry and angular dependence of IFX emission in the LH1 sample. 14
Figure S9: Temperature, Power and Polarisation resolved Integrated PL intensity of IFX in LH3 sample. 15
Figure S10: Excitation polarization-independent anisotropic IFX emission in LH3 sample. 16
Figure S11: HAADF images of J1, J2 and J3 junctions. 17
Figure S12: Raman spectra and peak position analysis across the sharp junction in LH1 sample. 18
Figure S12: Raman spectra and peak position analysis across the sharp junction in LH3 sample. 19
Figures S14–S18: Convergence tests for the first-principles calculations. 20
Table S1: Calculation parameters for the first-principles calculations. 25

Methods

Experimental Methods

Sample Fabrication

(i) Synthesis: The three-junction monolayer $MoSe_2$ -WSe_2 LHs were fabricated via a one-pot, water-assisted CVD process.[1] The flakes were grown on Si/SiO_2 substrates kept at a temperature of 780-800°C, while $MoSe_2$ and WSe_2 bulk precursors were placed at 1050° C in the upstream. By dynamically adjusting the carrier gas during growth, selective evaporation and deposition of each TMD layer was achieved. In the first stage, N_2 gas flowed through a water bubbler (200 s.c.c.m.) to oxidize the solid precursors, promoting the evaporation of Mo-based species to form $MoSe_2$. Subsequent switching to a reducing Ar+H_2 (5%) atmosphere enabled the rapid reduction of Mo-based oxides to Mo. While slow reduction of W-based oxides from their higher to lower sub-oxide phases maintain the evaporation of W-related sub-oxides, leading to WSe_2 growth under the same temperature on the edges of the pregrown $MoSe_2$ template. Repeating this gas-flow alternation enabled the formation of continuous multi-junction LHs with independent control over the lateral dimensions of each domain. The different oxidation and reduction rate of Mo and W-based sub-oxides resulted in the observed $MoSe_2$ to WSe_2 transition as smooth junction while WSe_2 to $MoSe_2$ as sharp junction.

(ii) Transfer: hBN flakes were mechanically exfoliated on PDMS (Gel-pack) and transferred onto the 285 nm SiO_2 /Si substrate. A water assisted delamination strategy was applied to pick the as grown TMD flakes on PDMS and subsequently transferred onto the desired hBN. Another thin hBN flakes were again mechanically exfoliated onto PDMS and transferred on top of the TMD/h-BN stack. The multilayer stacks were annealed under 10 -2 mbar vacuum for 2hrs. We fabricated two stacking samples with ∼5 nm (LH1 sample) and ∼30 nm (LH2 sample) thick of each bottom and top hBN.

Measurement Setup

The room- and low- temperature measurements were performed on Renishaw InVia Ra-

man setup. For room temperature measurements, a Leica 100X objective lens with a numerical aperture (NA) of 0.85 was used. For Low temperature measurements, the sample was placed in a Montana cryostat chamber and a Leica 50X objective with a NA of 0.5 was used. Low temperature scans were taken with the help of a bendy arm coupled with the microscope stage of the Renishaw system. All Raman and photoluminescence measurements were done by employing 2400 lines/mm and 600 lines/mm grating respectively. For all measurements, a green laser of wavelength 532 nm was employed.

First-principles Calculations

Ground state The calculations were performed with the Vienna Ab Initio Software Package (VASP),[2] using the Projector-Augmented Wave (PAW) method and recommended *GW* pseudopotentials with 14 (Mo and W) and 6 (Se) valence electrons, respectively. A simulation cell was used that contains a monolayer LH consisting of 12 formula units of $MoSe_2$ and 12 formula units of WSe_2 [see Figure 4 (a) in the main text]. Periodic boundary conditions in all three directions were employed. First, density functional theory (DFT) in the generalized-gradient approximation parametrised by Perdew, Burke, and Ernzerhof[3] (PBE) including spin-orbit coupling was employed to obtain the optimized atomic structure and an approximate electronic band structure. Plane-wave basis functions with energies up to 520 eV were used. Both the atomic positions and the cell parameters were optimized until the total-energy differences between consecutive iteration steps fell below 0.001 meV for the optimization of the electronic density and below 0.01 meV for the optimization of the atomic structure. The reciprocal unit cell was sampled with a mesh of $1\times24\times1$ k-points, equivalent to a $24 \times 24 \times 1$ k-point mesh for a single 3-atom monolayer unit cell of $MoSe_2$ or WSe_2.

Electronic structure An improved electronic band structure was obtained by means of many-body perturbation theory in the *GW* approximation.[4] A single *GW* iteration (G_0W_0) was performed. 2304 electronic bands were included in the *GW* calculation. All 624 valence

bands and the lowest 20 conduction bands were corrected with GW. An energy cutoff of 30 eV was employed for the dielectric matrix. The dielectric matrix was determined at 30 different frequencies.

Excitons The optical transitions were determined using the Bethe-Salpeter equation (BSE).[5] 20 occupied and 20 unoccupied bands were considered. Convergence tests were carried out to validate our choice of calculation parameters, see below. Only neutral excitons were considered.

The oscillator strengths $f(E)$ from the BSE, where E is the photon energy, were computed as

$$f(E) = \sum_{\lambda} f^{(\lambda)}\, \tilde{\delta}(E - E^{(\lambda)}) = \sum_{\lambda} \sum_{i=1}^{3} \left| \sum_{vc\mathbf{k}} A^{(\lambda)}_{vc\mathbf{k}} \langle v\mathbf{k}|x_i|c\mathbf{k}\rangle \right|^2 \tilde{\delta}(E - E^{(\lambda)}), \tag{1}$$

where $\mathbf{A}^{(\lambda)}$ is the coupling coefficient for the λth energy eigenvalue $E^{(\lambda)}$ of the BSE, so that $\sum_{v'c'\mathbf{k}'} \mathcal{H}^{\mathrm{BSE}}_{vc\mathbf{k},v'c'\mathbf{k}'} A^{(\lambda)}_{v'c'\mathbf{k}'} = E^{(\lambda)} A^{(\lambda)}_{vc\mathbf{k}}$,[6,7] and the excitonic wavefunction is $\Psi^{(\lambda)}_X(\mathbf{r}_e, \mathbf{r}_h) = \sum_{cv\mathbf{k}} A^{(\lambda)}_{cv\mathbf{k}} \psi^*_{v\mathbf{k}}(\mathbf{r}_h) \psi_{c\mathbf{k}}(\mathbf{r}_e)$,[6] where $\mathbf{r}_e$ and $\mathbf{r}_h$ are the positions of electron and hole. The indices v, c, and $\mathbf{k}$ denote valence bands, conduction bands, and k-points in the reciprocal unit cell. The x_i are cartesian coordinates, $\tilde{\delta}(E)$ is a broadened δ function. The oscillator strengths are closely related to the imaginary part of the dielectric permittivity and the absorption spectrum. The spectrum of the heterostructure was red-shifted *a posteriori* by 0.09 eV (the energy difference between the brightest excitons in $MoSe_2$ and the heterostructure) to correct for quantum confinement in the heterostructure arising from the small lateral extension of the simulation box.

Charge transfer, dipole moment To visualize the spatial distribution of excited electron and hole, we define $\varrho^{(\lambda)}_{e/h}$ for each excitonic state λ as

$$\varrho^{(\lambda)}_e \sim \sum_{vc\mathbf{k}} |A^{(\lambda)}_{vc\mathbf{k}}|^2\, |\psi_{c\mathbf{k}}|^2 \text{ and } \varrho^{(\lambda)}_h \sim \sum_{vc\mathbf{k}} |A^{(\lambda)}_{vc\mathbf{k}}|^2\, |\psi_{v\mathbf{k}}|^2. \tag{2}$$

This quantity is not an observable. It is related, but not identical to a conditional probability density. It is used here because other than the conditional probability density, its evaluation requires only negligible computational resources.
Charge transfer was defined as

$$\mathrm{CT} = \int\limits_{\mathrm{WSe_2}} \mathrm{d}x \, (\varrho_h - \varrho_e), \tag{3}$$

with $-1 \leq \mathrm{CT} \leq 1$ and $\mathrm{CT} > 0$ if the hole is predominantly on the WSe_2 side and the electron on the $MoSe_2$ side. We label an IFX as "$\mathrm{IFX_{CT}}$" if CT> 0.4, as "$\mathrm{IFX_{MoSe_2}}$" if more than 50% of both electron and hole are on the $MoSe_2$ side, and analoguous for $\mathrm{IFX_{WSe_2}}$. The dipole moment μ is computed as

$$\mu = |e| \int\limits_{\mathrm{LH}} \mathrm{d}x \, x \, (\varrho_h - \varrho_e), \tag{4}$$

with $\mu > 0$ if the positive charge is on WSe_2 and the negative on $MoSe_2$. The dipole moment μ is not identical to the dipole matrix element

$$\mathbf{d} = -|e| \int \psi_e^* \, (\mathbf{r}) \, \mathbf{r} \, \psi_h(\mathbf{r}), \tag{5}$$

also called transition dipole moment or transition moment integral, which determines the polarization of emitted light.

Dielectric screening by substrate and encapsulation In the calculation we do not directly consider encapsulation with boron nitride, since this would further increase the already large computational load. Instead we vary the dielectric permittivity ε by varying the distance between periodic images of the LH.

Additional results from microscopy and spectroscopy

S1: Optical images of non-encapsulated and hBN encapsulated $MoSe_2$-WSe_2 lateral heterostructures

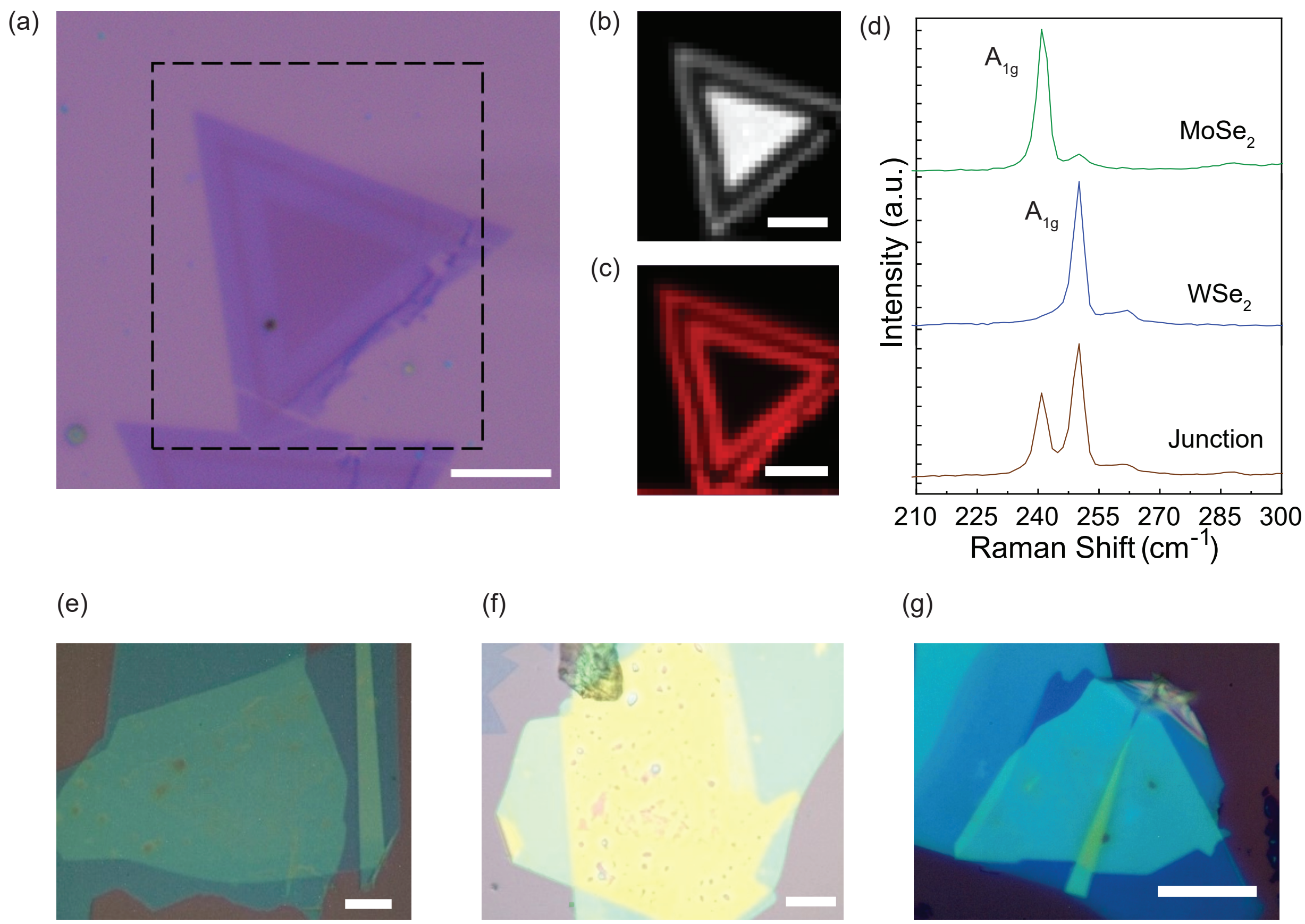

Figure S1: Optical images of lateral heterostructures (LHs). (a) Optical image of non-encapsulated $MoSe_2$-WSe_2 LH grown by chemical vapor deposition on SiO_2/Si substrate, with alternating $MoSe_2$ (dark contrast) and WSe_2 (bright contrast) (b),(c) Raman maps corresponding to $MoSe_2$ (248 cm^{-1}) and WSe_2 248 (cm^{-1}) respectively. (d) Raman spectra of $MoSe_2$, WSe_2 and junction. (e),(f) Optical images of hBN encapsulated LH samples: LH1, LH2 and LH3. Scale bar: (a),(b),(c),(e) 5μm and (f),(g) 20μm

S2: Room temperature characterisation of LH2 sample

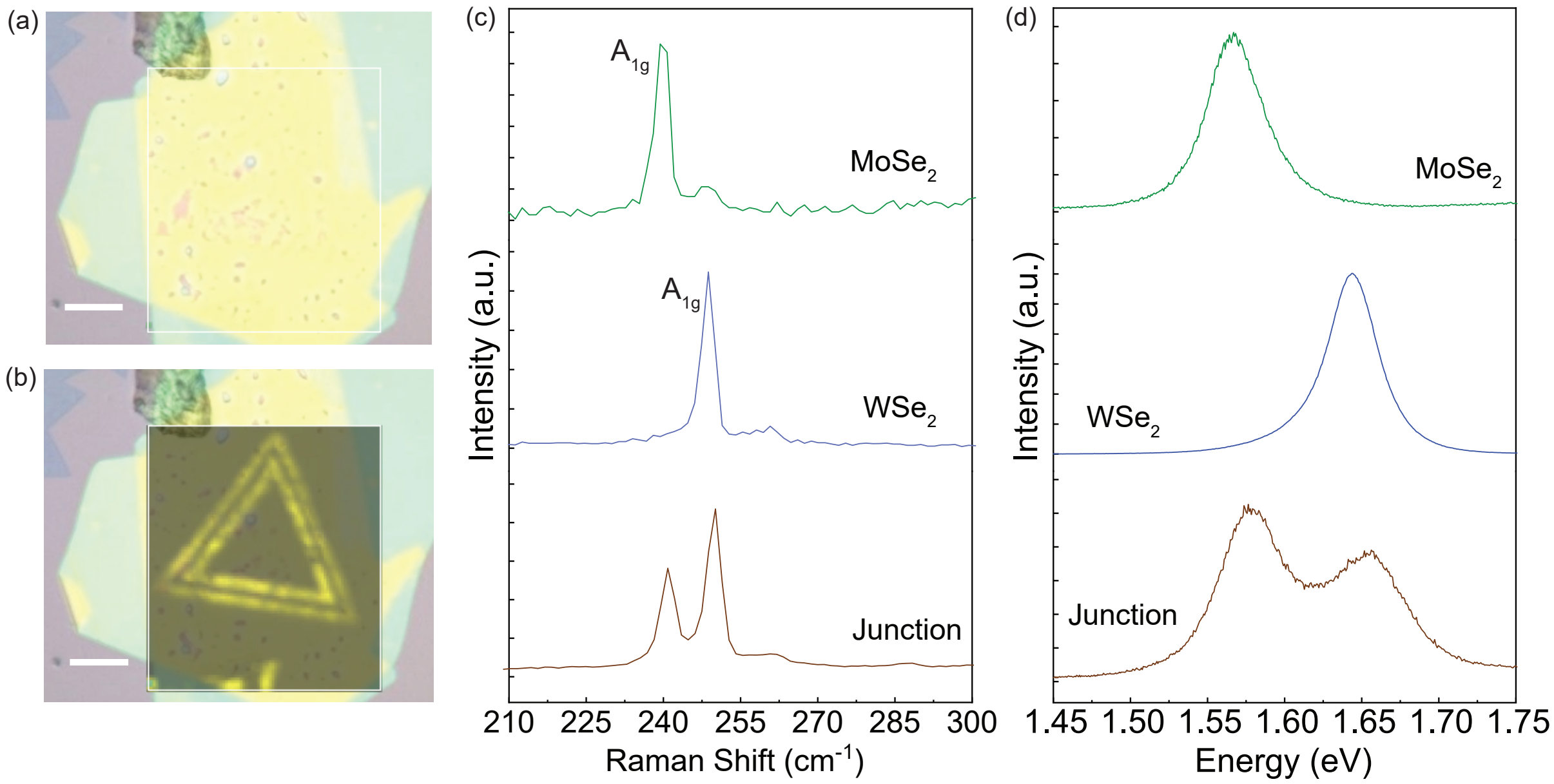

Figure S2: Room temperature measurements of LH2 sample. (a) Optical image of LH encapsulated in hBN. (b) Raman map at resonant Raman shift 248 cm^{-1} overlapped on the optical image of the hBN encpsulated LH. Scale bar (a),(b) 20μm (c) Raman spectra from different regions of the LH. (d) PL spectra fom different regions of LH.

S3: Low temperature results of LH2 sample

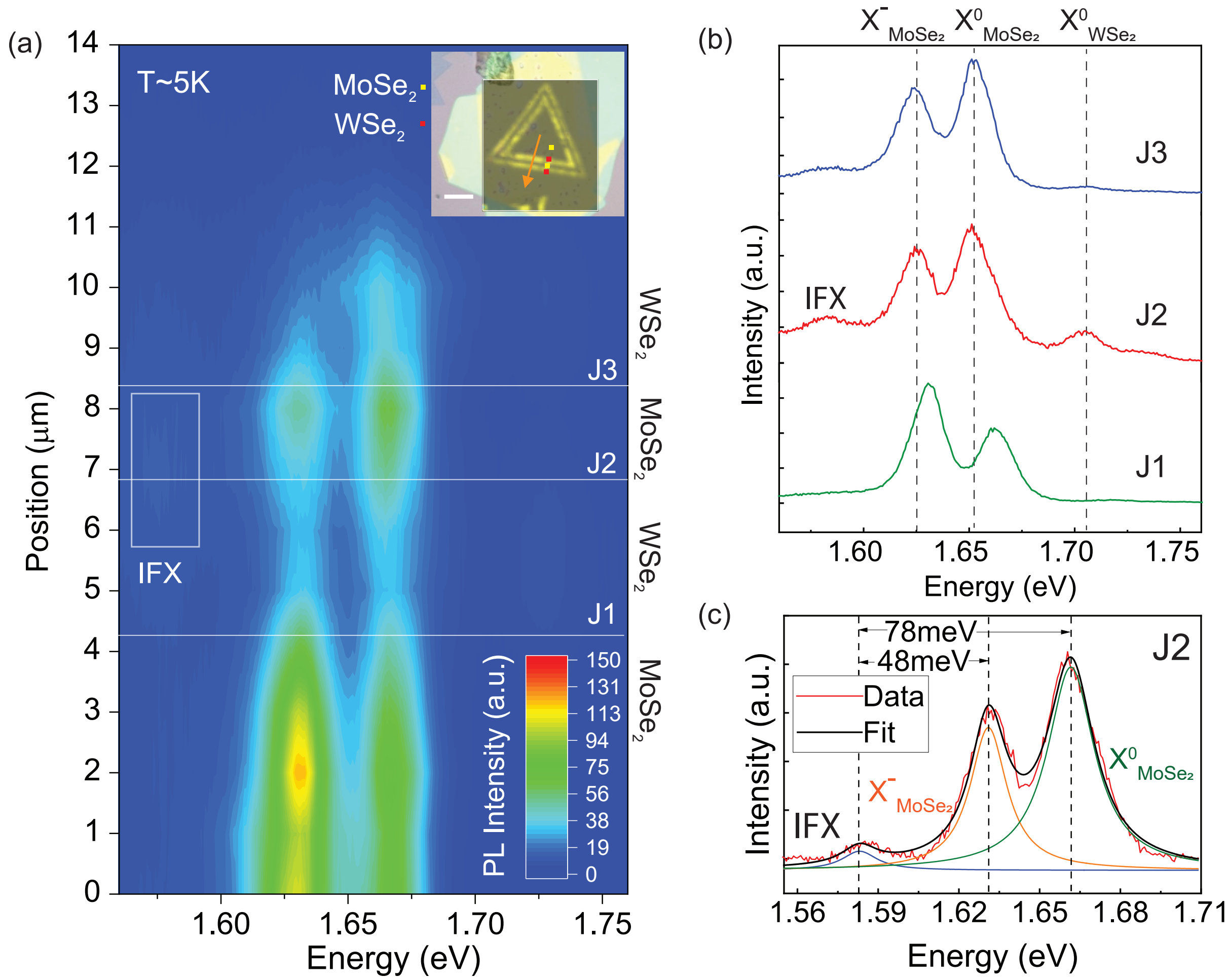

Figure S3: Low-temperature measurements of LH2 (a) Contour map of hBN encapsulated $MoSe_2$-WSe_2 lateral heterostructure at T∼5K, excited with a 532nm CW laser at a power of 3 μW. Apart from low-temperature excitonic and trionic peaks of $MoSe_2$ and WSe_2, an additional peak (∼1.585eV) is observed at the sharp junction J2 only, shown by white rectangle in the figure. This peak is absent at the smooth junctions J1 and J3. The Orange arrow in the inset shows the direction of the line scan from which the contour map is plotted. Inset scale bar 20μm. (b) PL spectra from the three junctions at 5K. (c) PL spectrum from junction J2 fitted with Lorentzian functions. The interface exciton, the $MoSe_2$ A exciton, and the $MoSe_2$ trion are marked as IFX, $X^0_{\mathrm{MoSe_2}}$, and $X^T_{\mathrm{MoSe_2}}$, respectively. The IFX peak energy lies 48 meV below the trion and 78 meV below the exciton peak of $MoSe_2$, and has a linewidth of 15.9 meV.

S4: Low temperature results of LH3 sample

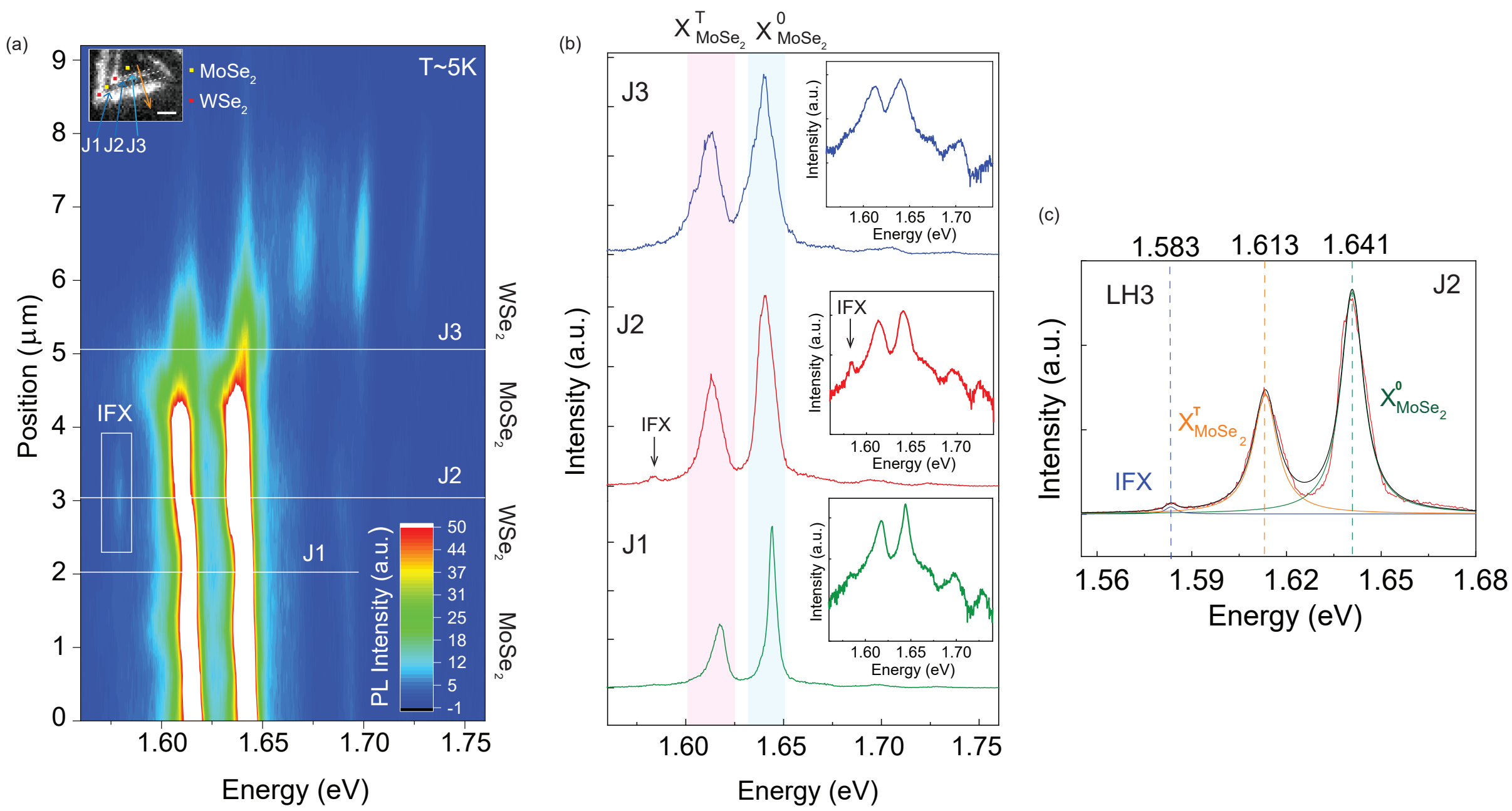

Figure S4: Low-temperature measurements of LH3 (a) Contour map of hBN encapsulated $MoSe_2$-WSe_2 lateral heterostructure at T∼5K. An additional peak (∼1.58eV) is observed at the sharp junction J2 only, shown by white rectangle in the figure. This peak is absent at the smooth junctions J1 and J3. The Orange arrow in the inset shows the direction of the line scan from which the contour map is plotted. Inset scale bar 5μm. (b) PL spectra from the three junctions at 5K. The corresponding insets show the same spectra plotted on a logarithmic intensity scale to highlight the weak IFX emission. (c) (c) PL spectrum from junction J2 fitted with Lorentzian functions. The IFX peak energy lies 30 meV below the trion and 58 meV below the exciton peak of $MoSe_2$, and has a linewidth of 4.9 meV.

S5: Temperature-dependent and Power-dependent data of LH1 sample

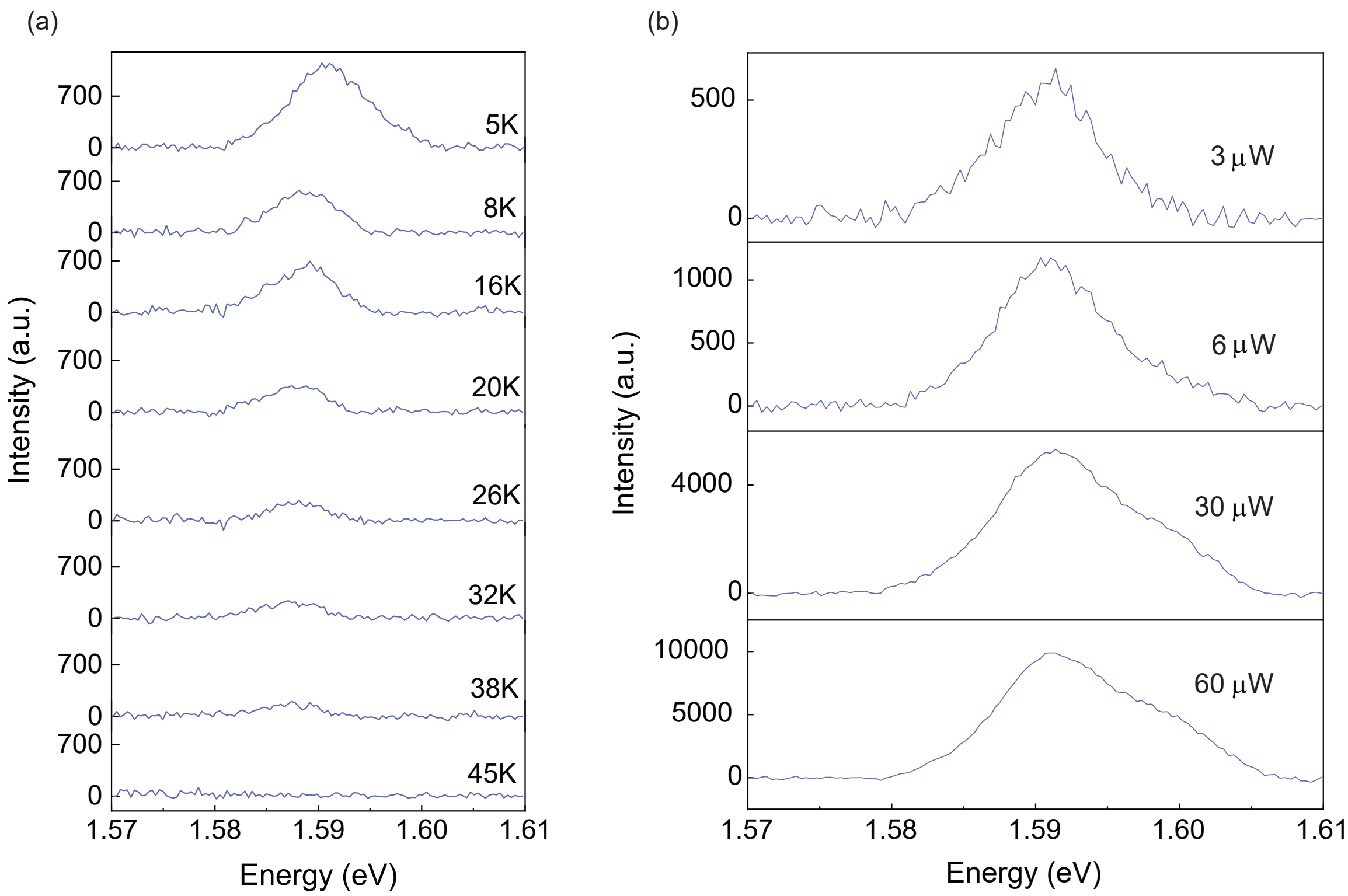

Figure S5: (a) Temperature-dependent PL spectra of the LH1 sample (b) Excitation power-dependent PL spectra of LH1 sample.

S6: Polarization-dependent data of LH1 sample

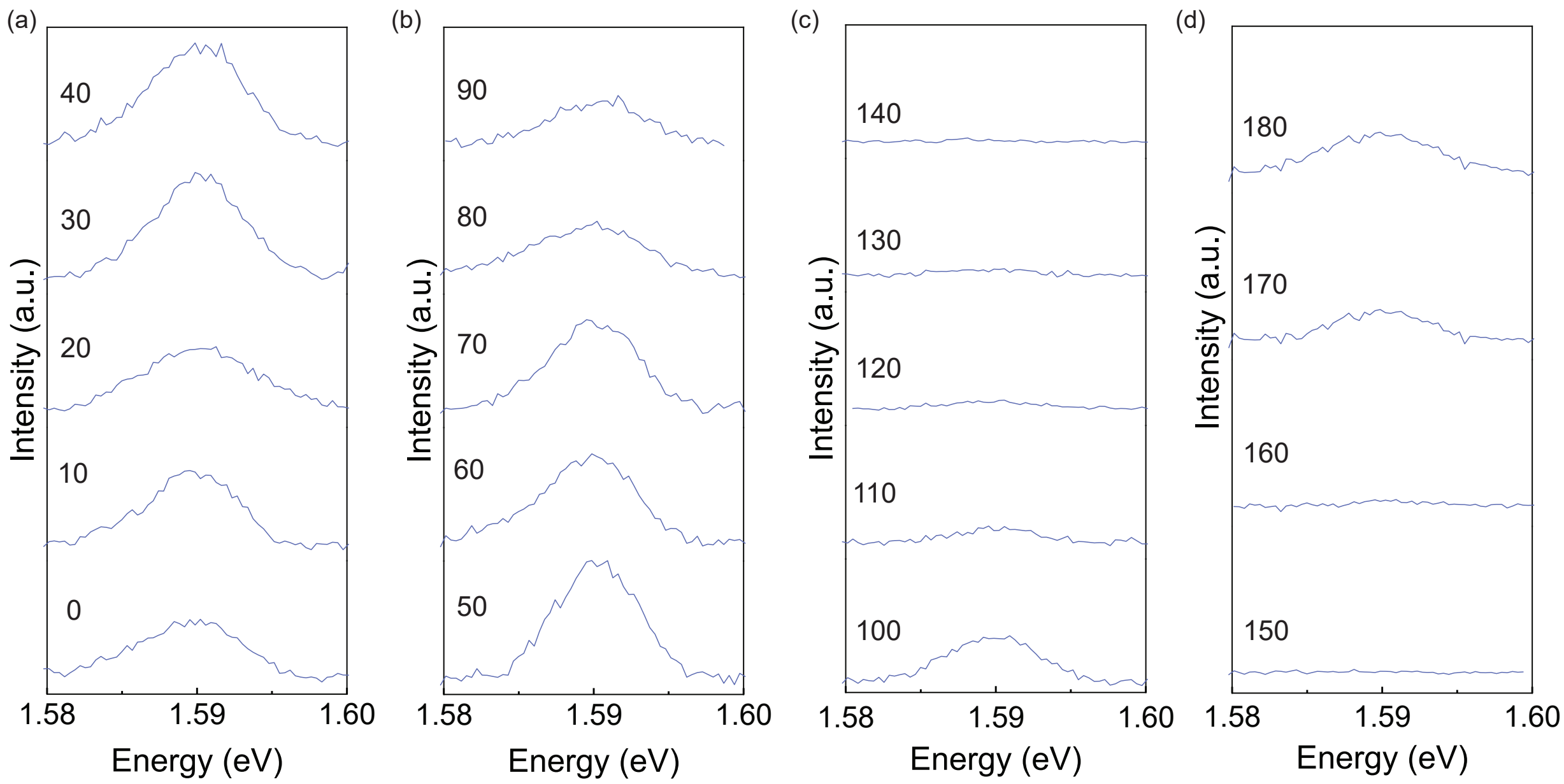

Figure S6: Polarization-resolved PL spectra of the LH1 sample recorded at different analyzer angles in the detection path.

S7: Additional analysis of IFX observed in LH1 sample

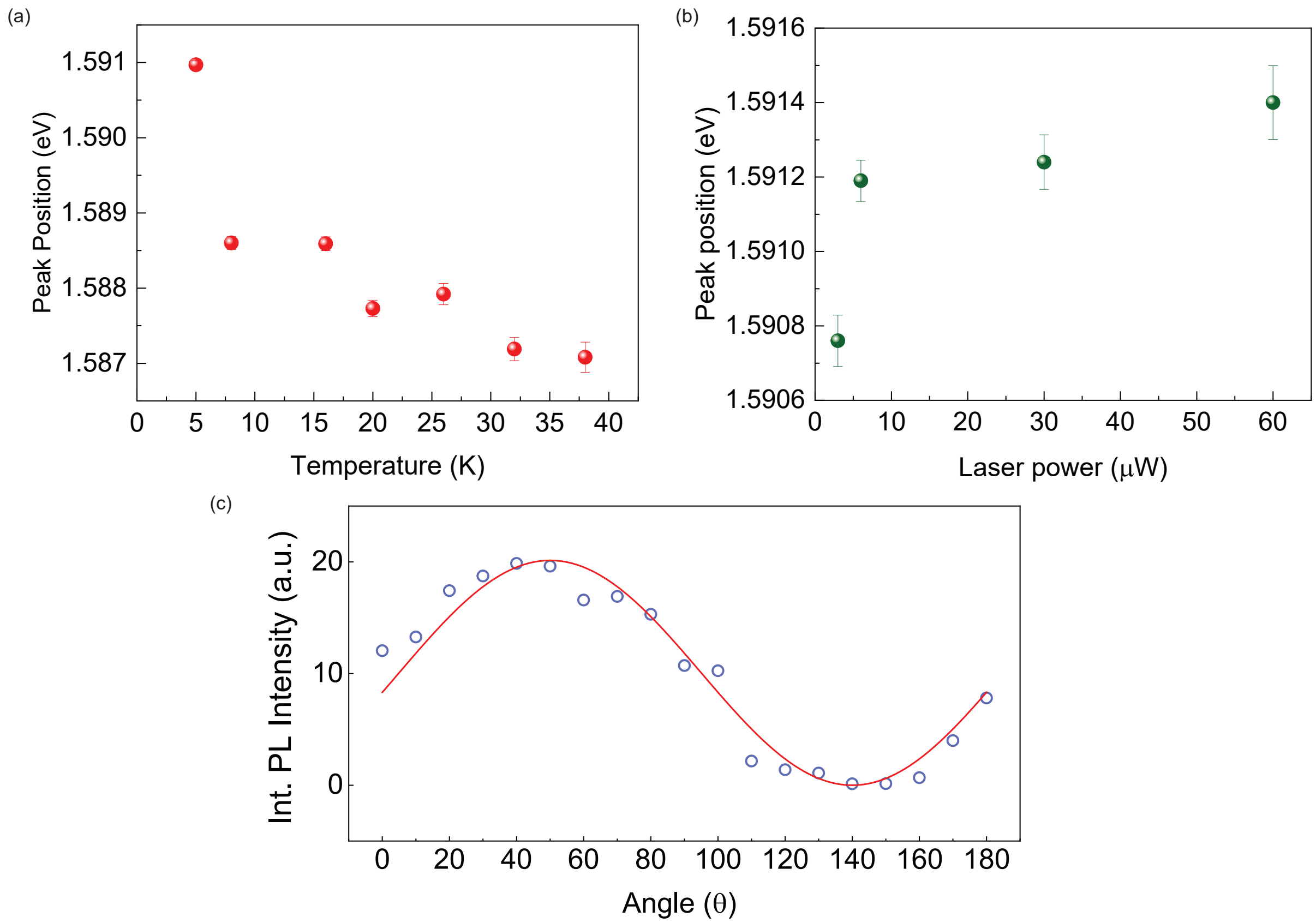

Figure S7: Temperature-dependent(a) and Excitation power-dependent(b) variation of the IFX peak position in the LH1 sample. (c) Integrated PL intensity of the IFX emission as a function of analyzer angle, shown in Cartesian coordinates. The data is well fitted with a cosine function, indicating a fixed dipole orientation of the emission.

S8: Polarization-resolved PL measurement geometry and angular dependence of IFX emission in the LH1 sample

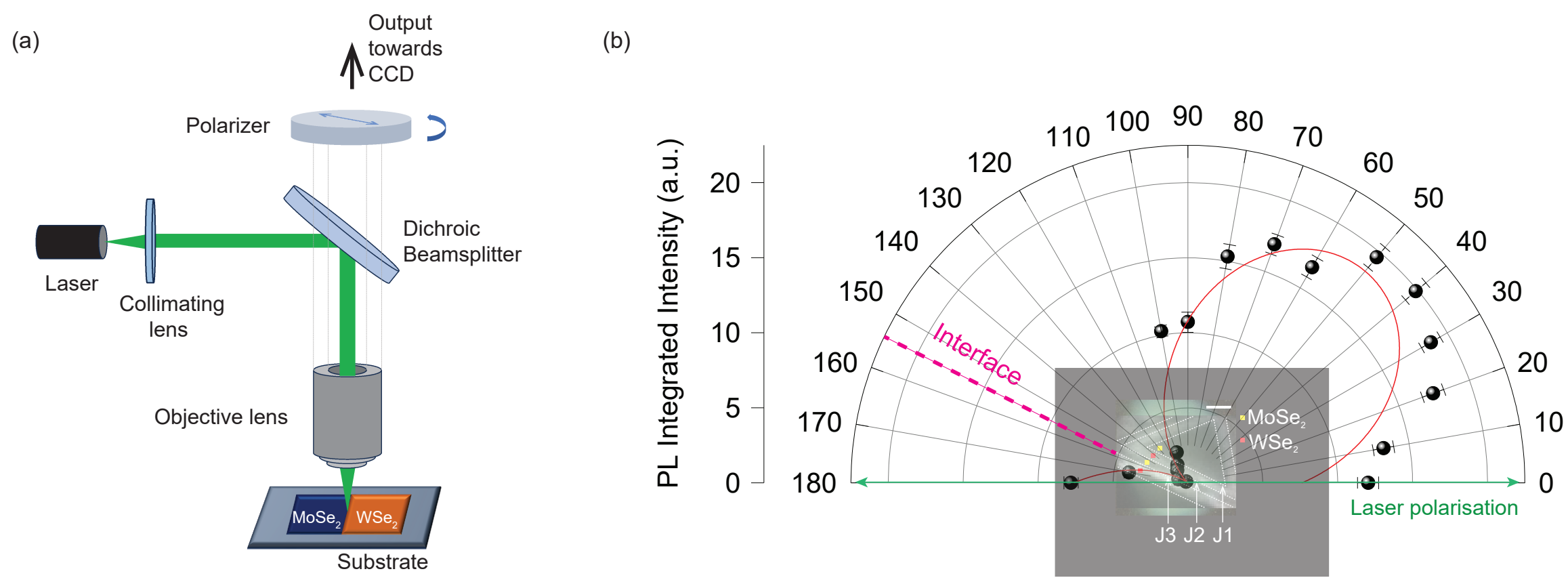

Figure S8: (a) Schematic of the polarization-resolved PL measurement set up in backscattering geometry with a rotatable analyzer placed in the detection path before the CCD. (b) Polar plot of the integrated PL intensity of IFX from LH1 sample. The magenta dashed line marks the interface, while the double arrowed green solid line denotes the excitation laser polarization. The optical image overlaid on the polar plot shows that the IFX emission is polarized nearly perpendicular to the interface.

Measurement set up: As shown in Fig. S8(a), the excitation laser passes through a half-wave plate (HWP) and a dichroic beamsplitter before being focused onto the lateral heterojunction through the objective lens. The PL signal emitted from the sample is collected in a backscattering configuration by the same objective and directed toward the CCD detector. A rotatable linear polarizer(analyzer) placed in the detection path is used to analyze the polarization-resolved PL response. Thus, the observed polarization dependence arises from the emitted PL signal itself rather than from any polarization bias introduced by the optical setup.

S9: Temperature, Power and Polarisation resolved Integrated PL intensity of IFX in LH3 sample.

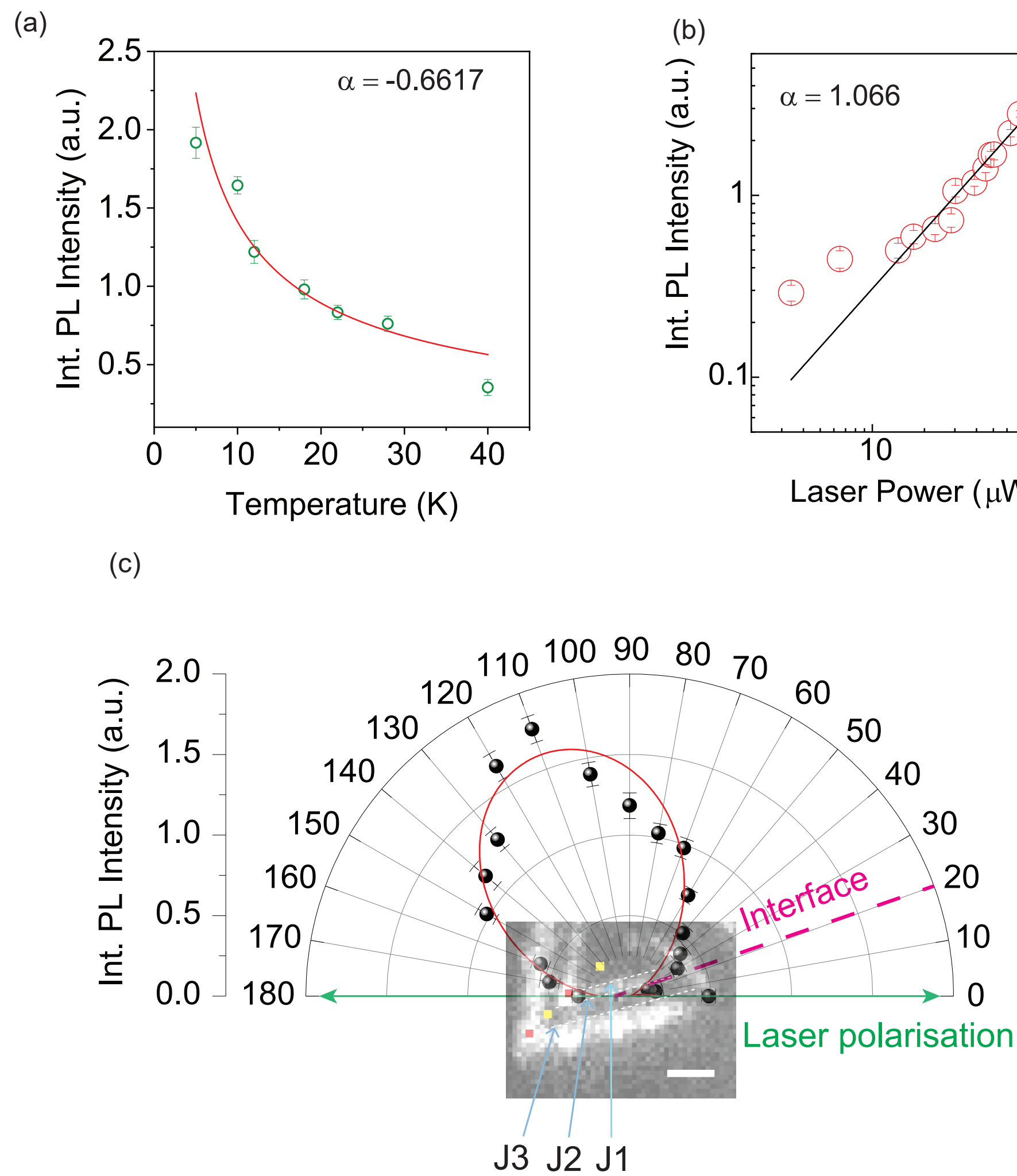

Figure S9: (a) Temperature-dependent and (b) Excitation power dependent integrated PL intensity of IFX in LH3 sample, fitted using a power-law relation. $I = AP^{\alpha}$ (c) Polarization-resolved integrated IFX PL intensity showing the angular dependence of the emission. The magenta dashed line marks the interface orientation, while the green solid line indicates the excitation laser polarization direction. The maximum PL intensity is observed along a direction nearly perpendicular to the interface. Optical image is overlaid with the polar plot. Scale bar: 5μm.

S10: Excitation polarization-independent anisotropic IFX emission in LH3 sample

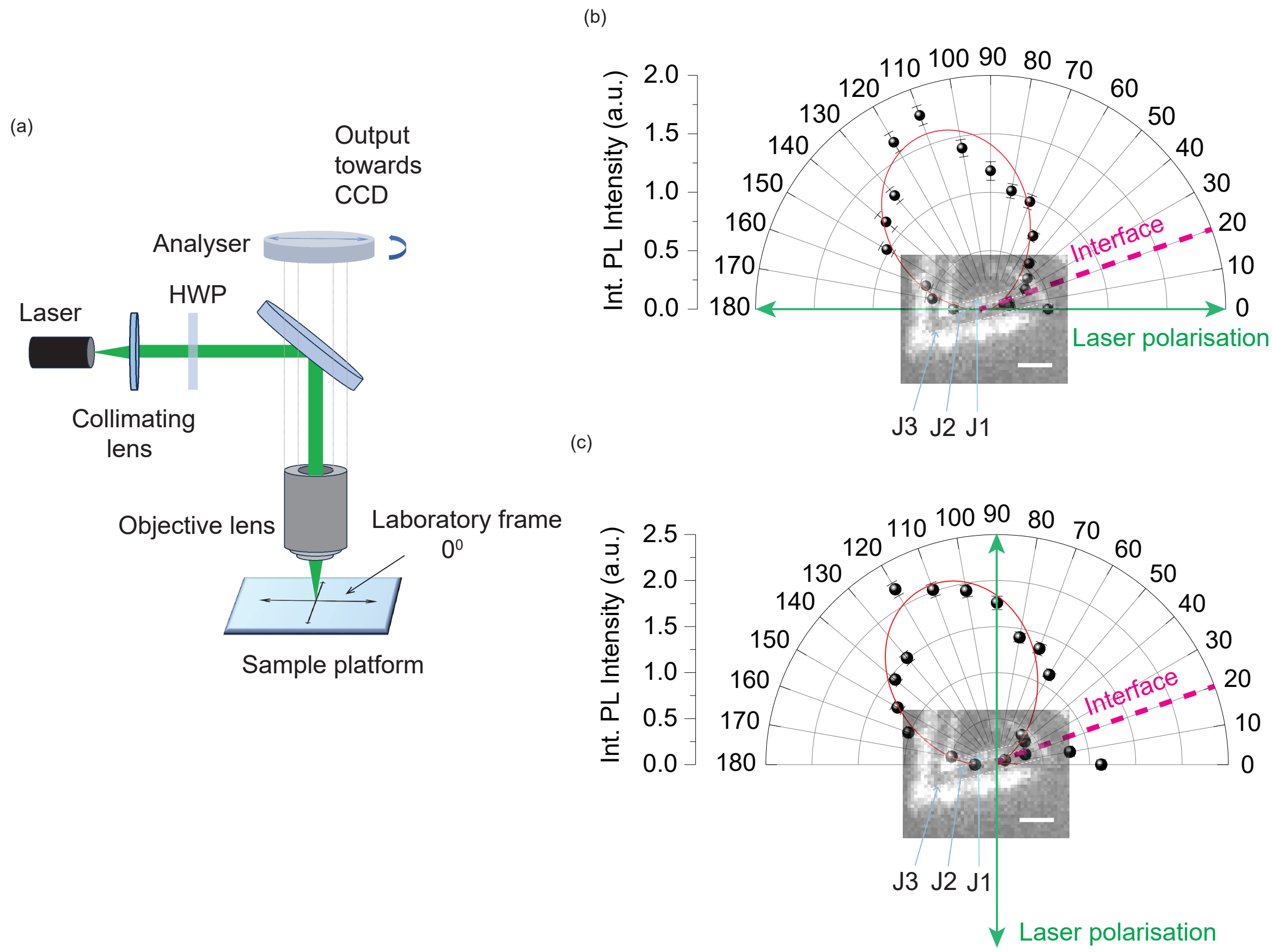

Figure S10: (a) Schematic of the polarization-resolved PL measurement setup, where the incident laser polarization is changed using a half-wave plate (HWP).(b,c) Polarization-resolved integrated PL intensity of the IFX from LH3 sample as a function of analyzer angle for horizontal and vertical excitation laser polarization directions, respectively. The excitation polarization was rotated using a half-wave plate in the input path. In both cases, the maximum PL intensity remains oriented nearly perpendicular to the interface (magenta dashed line), indicating that the IFX polarization is independent of the excitation laser polarization (double arrowed solid green line).

S11: HAADF images of J1, J2 and J3 junctions

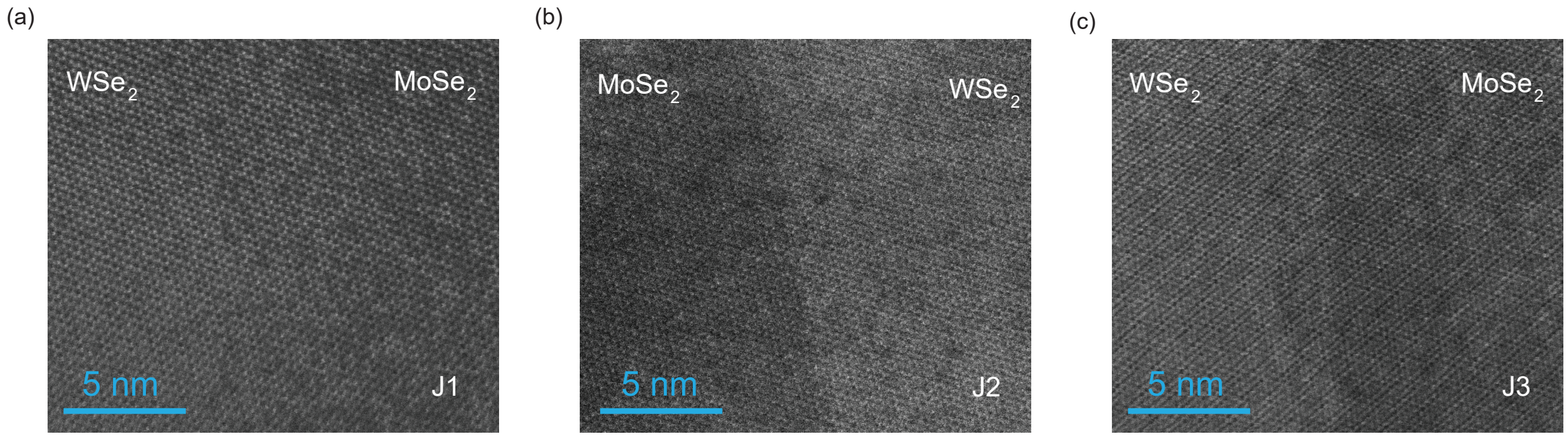

Figure S11: High-angle annular dark-field (HAADF) images of J1(diffuse), J2(sharp) and J3(intermediate) junctions

S12: Raman spectra and peak position analysis across the sharp junction in LH1 sample.

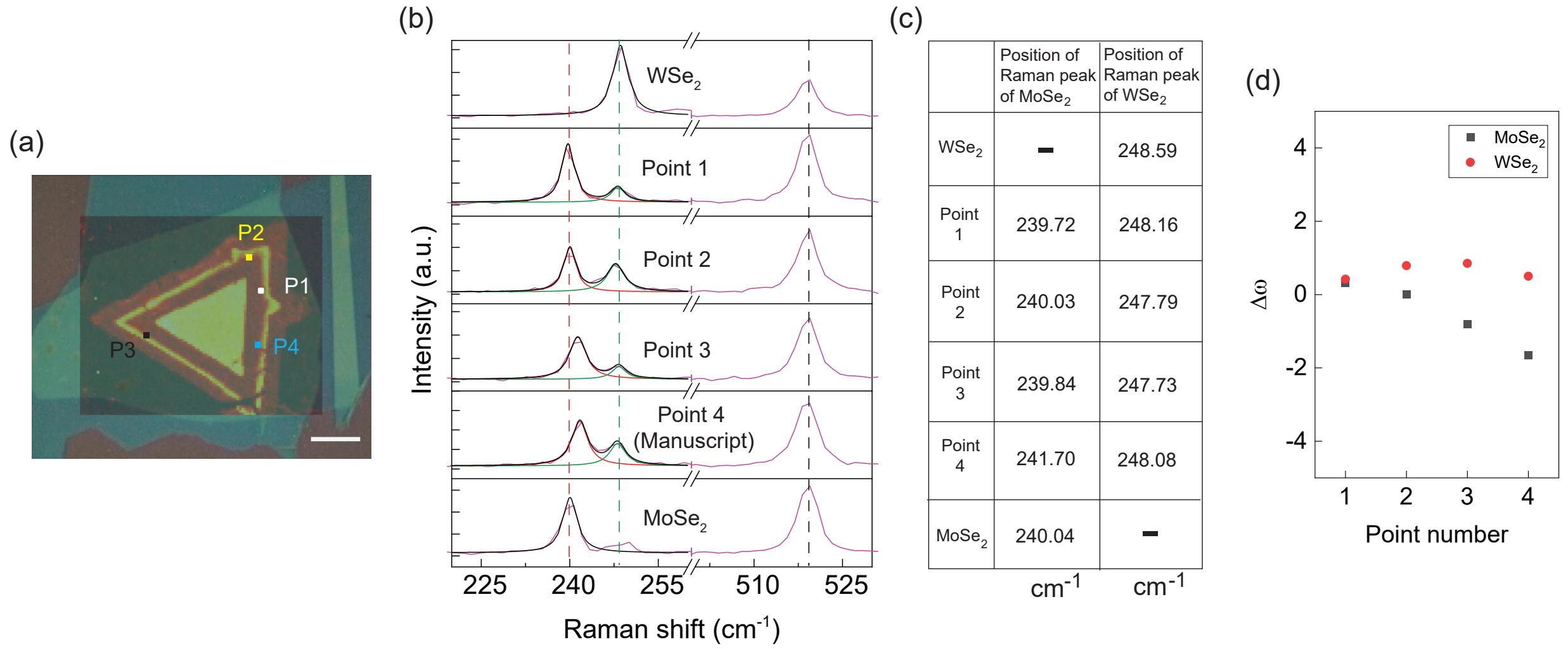

	Position of Raman peak of $MoSe_2$	Position of Raman peak of WSe_2
WSe_2	–	248.59
Point 1	239.72	248.16
Point 2	240.03	247.79
Point 3	239.84	247.73
Point 4	241.70	248.08
$MoSe_2$	240.04	–
	cm^{-1}	cm^{-1}

Figure S12: (a) Raman map overlaid with the optical image of LH1 sample. Scale bar: 10 μm. Points P(1-4) are the points on sharp junction J2, from where Raman spectra are taken and are shown in (b). (c) Comparative summary of Raman peak positions for $MoSe_2$ and WSe_2 modes measured at Points 1–4 near the sharp junction, referenced against pristine monolayer regions, highlighting only minor spatial variations in peak energies. (d) Variation in the Raman peak shift ($\Delta\omega$) of $MoSe_2$ and WSe_2 measured at four different sharp junction points with respect to their corresponding pristine regions. **The extracted $\Delta\omega$ values exhibit a standard deviation of 0.887 and 0.211 for $MoSe_2$ and WSe_2, respectively.**

S13: Raman spectra and peak position analysis across the sharp junction in LH3 sample.

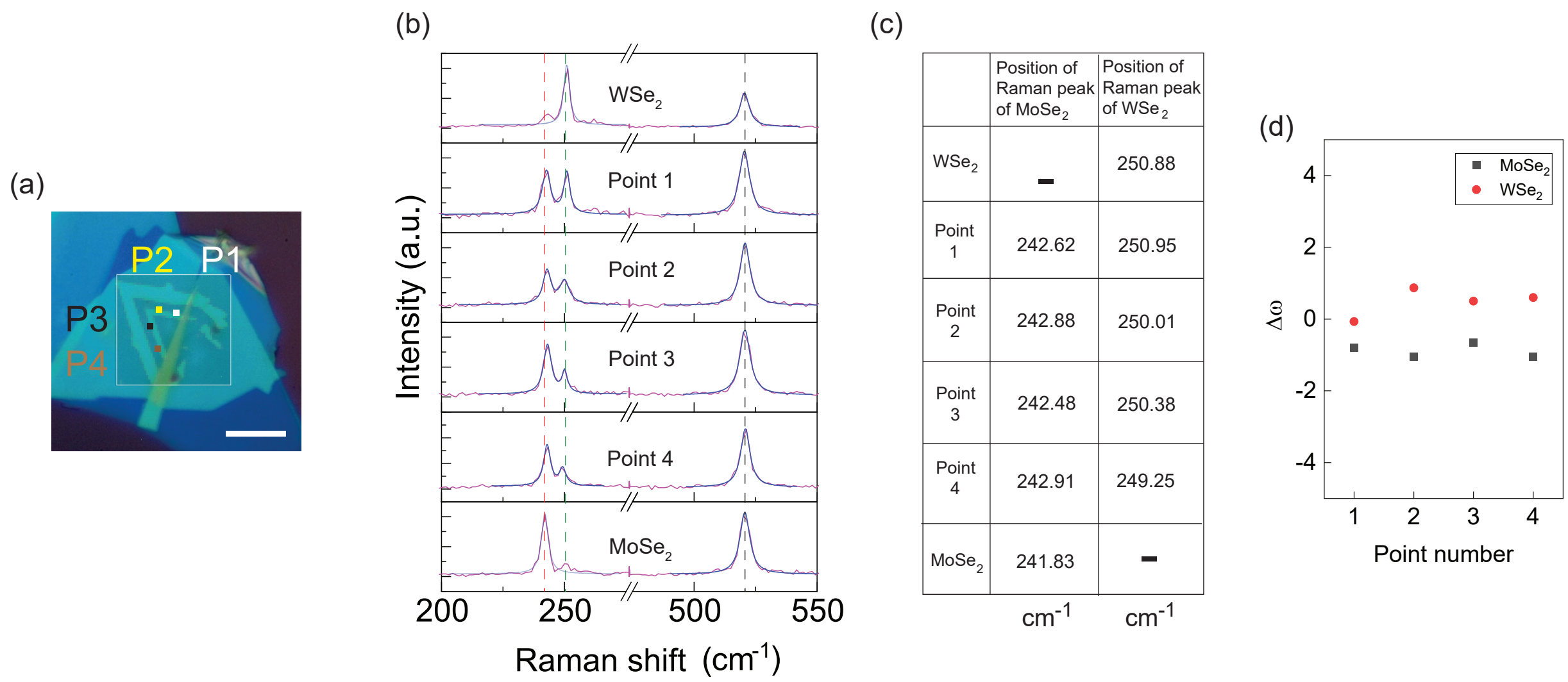

	Position of Raman peak of $MoSe_2$	Position of Raman peak of WSe_2
WSe_2	–	250.88
Point 1	242.62	250.95
Point 2	242.88	250.01
Point 3	242.48	250.38
Point 4	242.91	249.25
$MoSe_2$	241.83	–
	cm^{-1}	cm^{-1}

Figure S13: (a) Raman map overlaid with the optical image of LH3 sample. Scale bar: 5 μm. Points P(1-4) are the points on sharp junction J2, from where Raman spectra are taken and are shown in (b). (c) Comparative summary of Raman peak positions for $MoSe_2$ and WSe_2 modes measured at Points 1–4 near the sharp junction, referenced against pristine monolayer regions, highlighting only minor spatial variations in peak energies. (d) Variation in the Raman peak shift ($\Delta\omega$) of $MoSe_2$ and WSe_2 measured at four different sharp junction points with respect to their corresponding pristine regions. **The extracted $\Delta\omega$ values exhibit a standard deviation of 0.198 and 0.395 for $MoSe_2$ and WSe_2, respectively.**

Convergence tests for first-principles calculations

Number of bands in the GW calculation

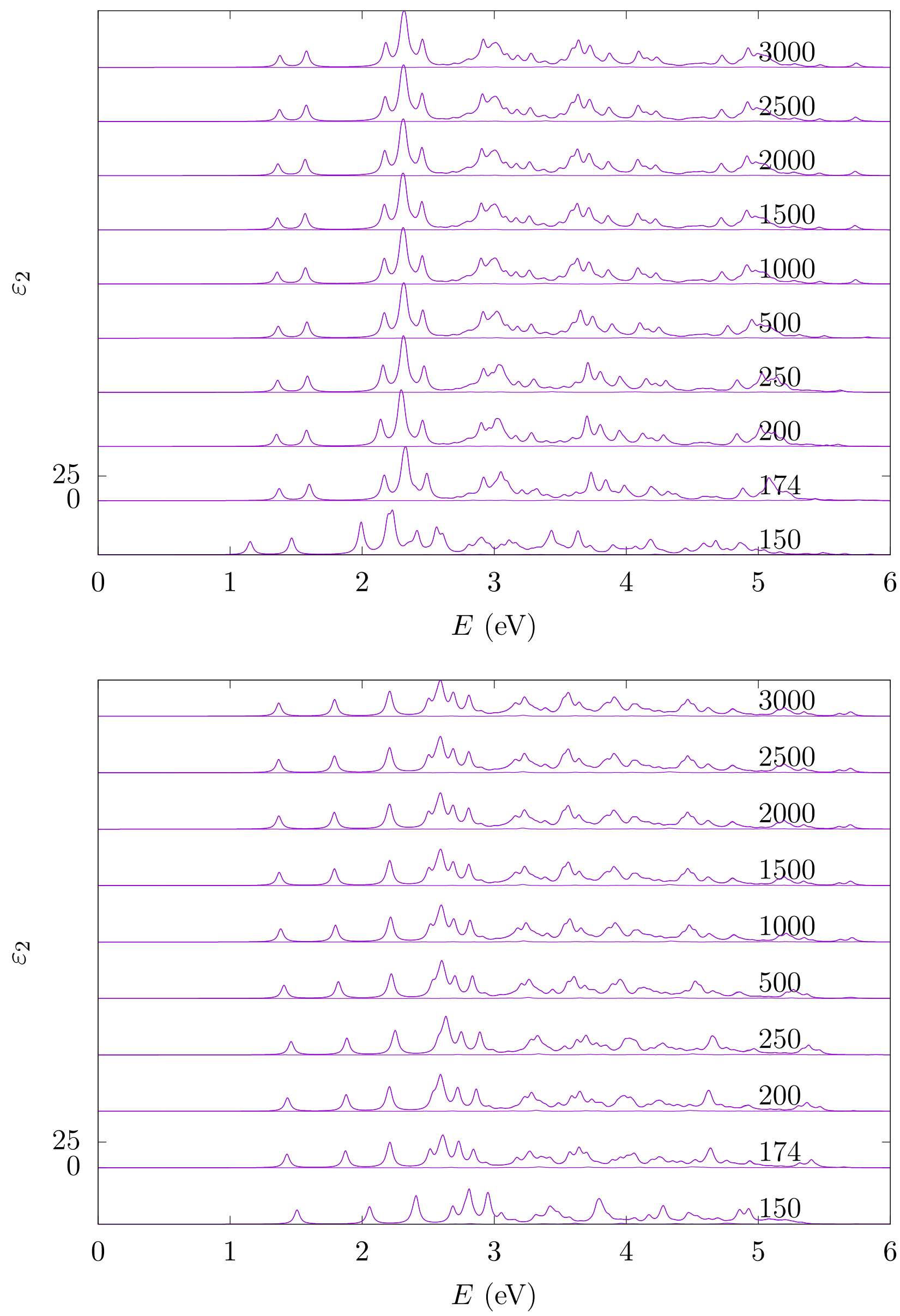

Figure S14: ε_2 of a $MoSe_2$ (top) and WSe_2 (bottom) bilayer as a function of the total number of bands NBANDS per 2 f.u. $MoSe_2$ or WSe_2 (per bilayer unit cell). At 174 bands, the bilayer spectrum is nearly converged, this corresponds to 2088 bands for the lateral HS with 12 f.u. $MoWSe_4$.

Number of bands in the BSE

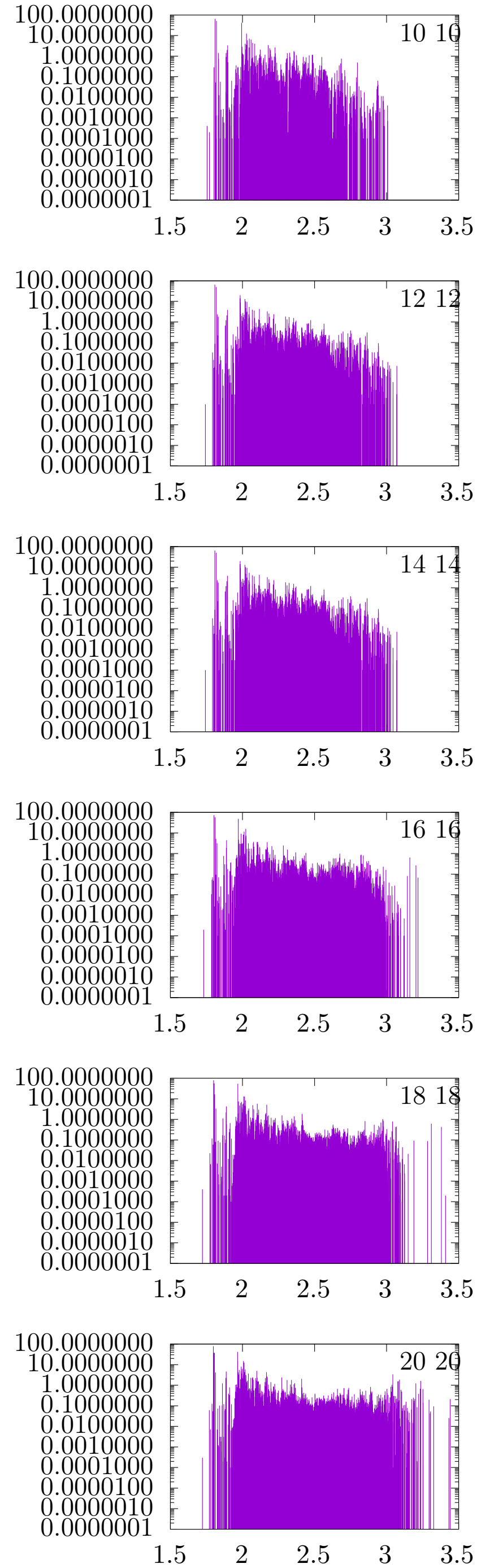

Figure S15: Excitonic oscillator strength (arb. units) vs. photon energy (eV) of the lateral $MoWSe_4$ heterostructure as a function of the number of valence and conduction bands in the BSE (“NBANDSO”, “NBANDSV”)

Plane-wave cutoff for the screening matrix

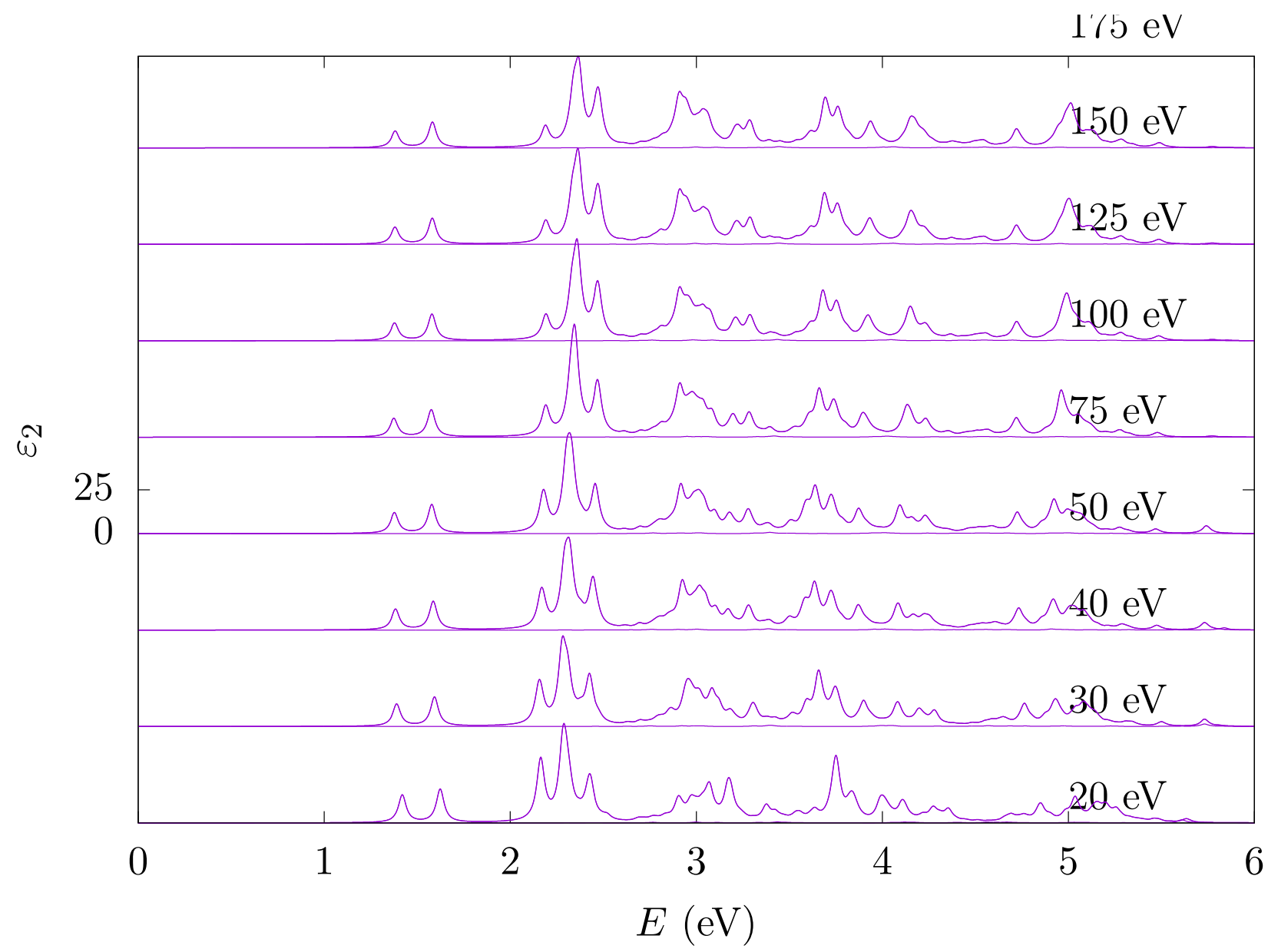

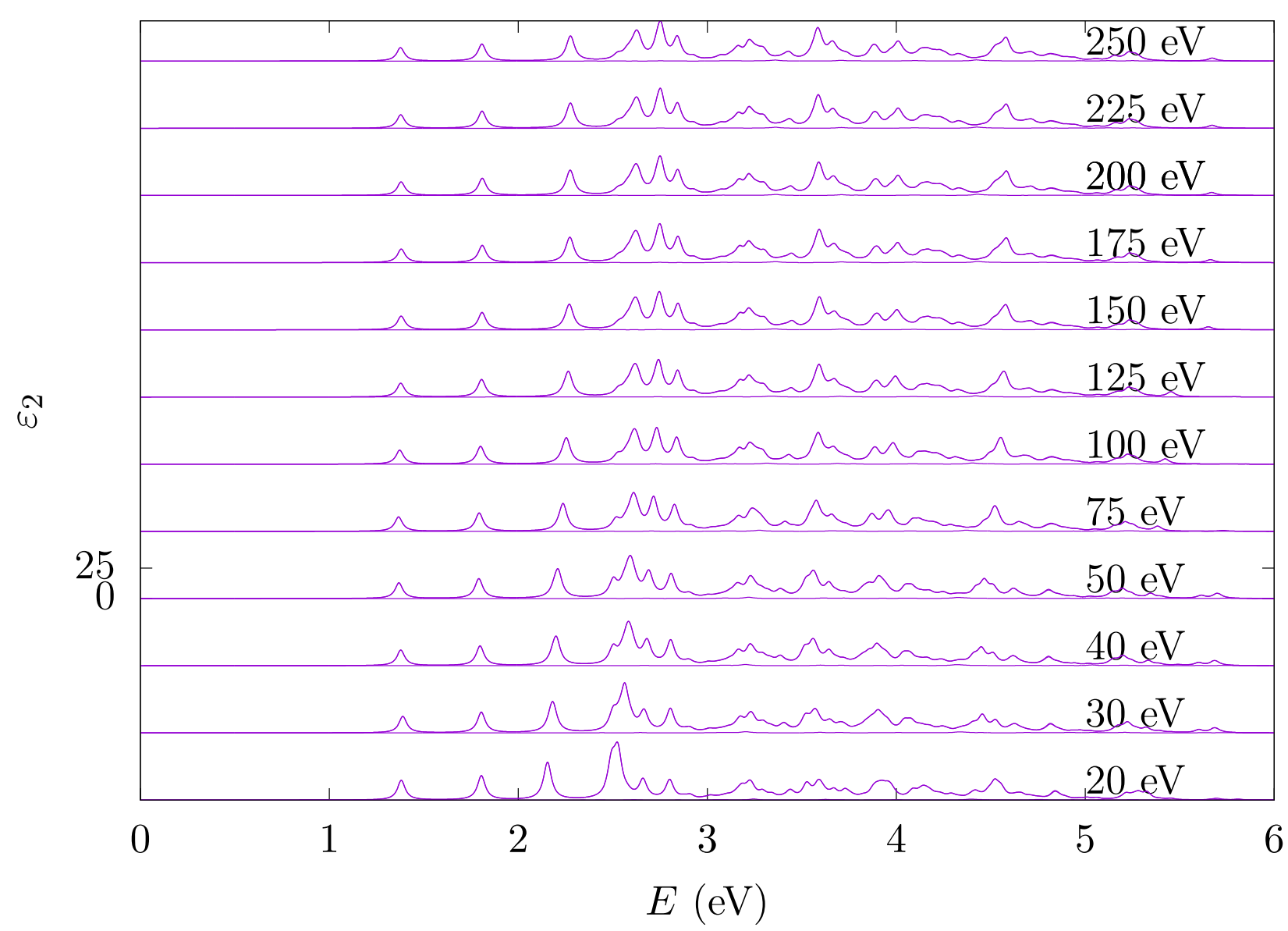

Figure S16: ε_2 of a $MoSe_2$ (top) and WSe_2 (bottom) bilayer as a function of the kinetic energy cutoff in the screening matrix ("ENCUTGW")

Number of frequencies for the screening matrix

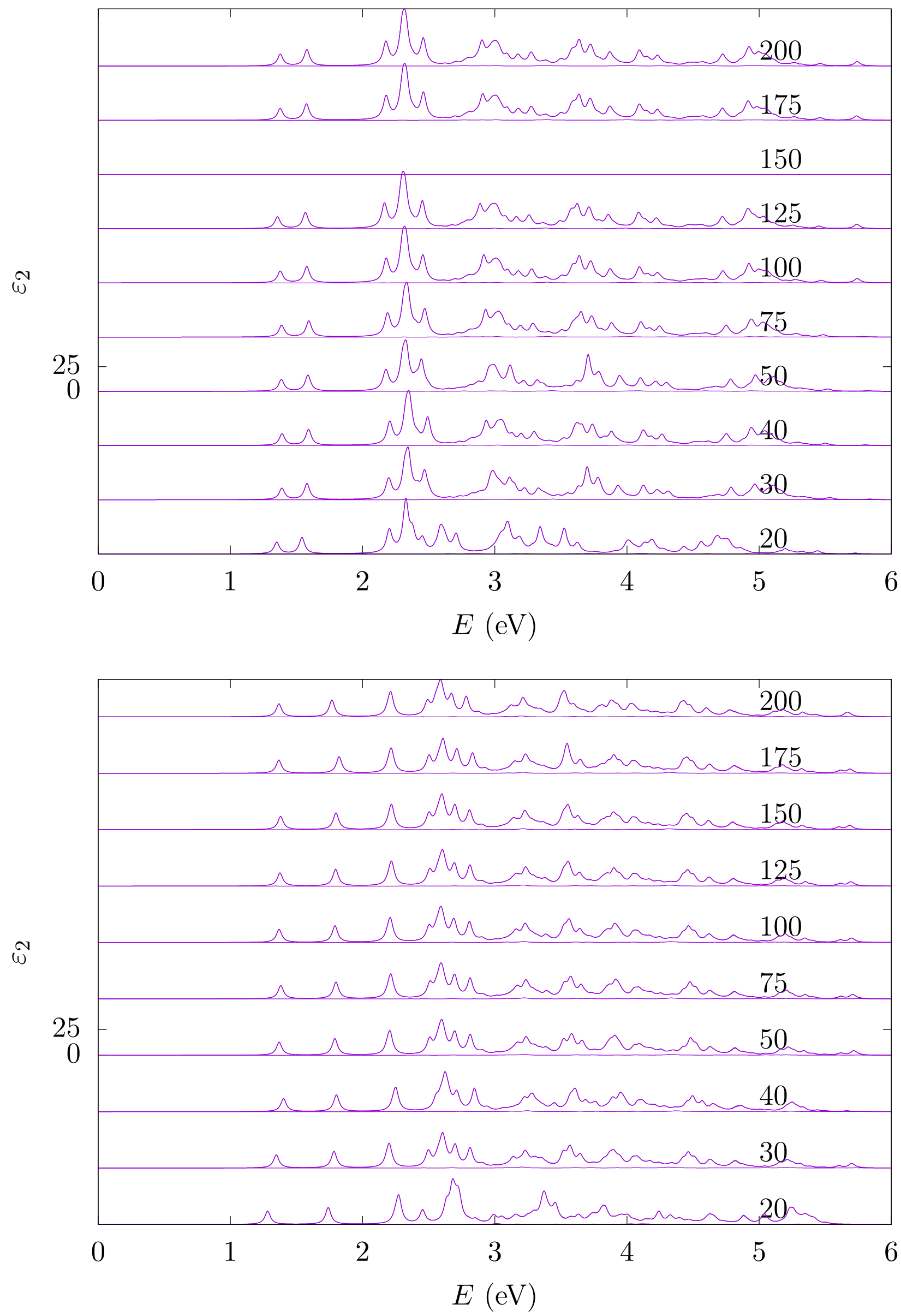

Figure S17: ε_2 of a $MoSe_2$ (top) and WSe_2 (bottom) bilayer as a function of the number of frequencies in the screening matrix ("NOMEGA")

Number of kpoints NKPOINTS

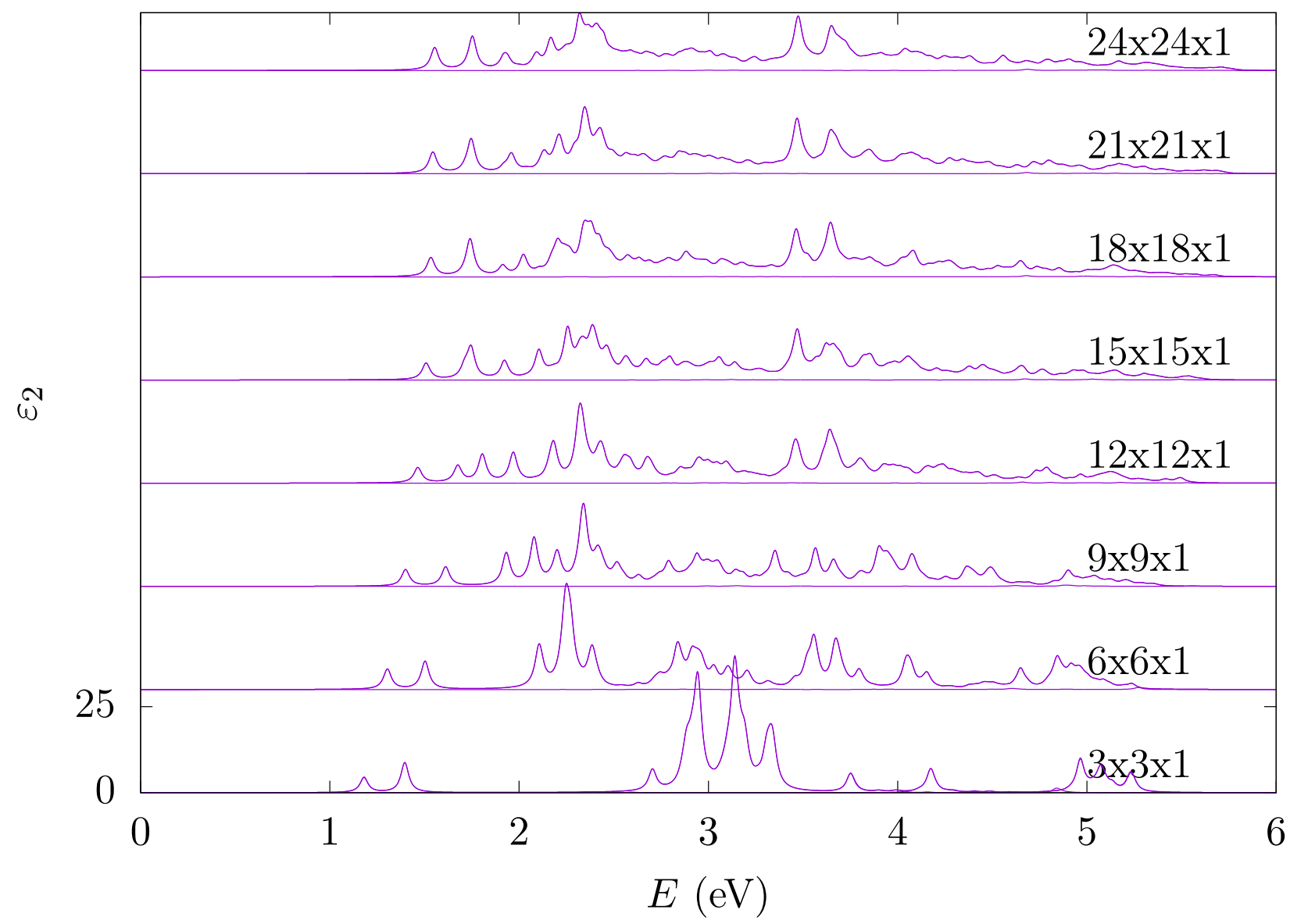

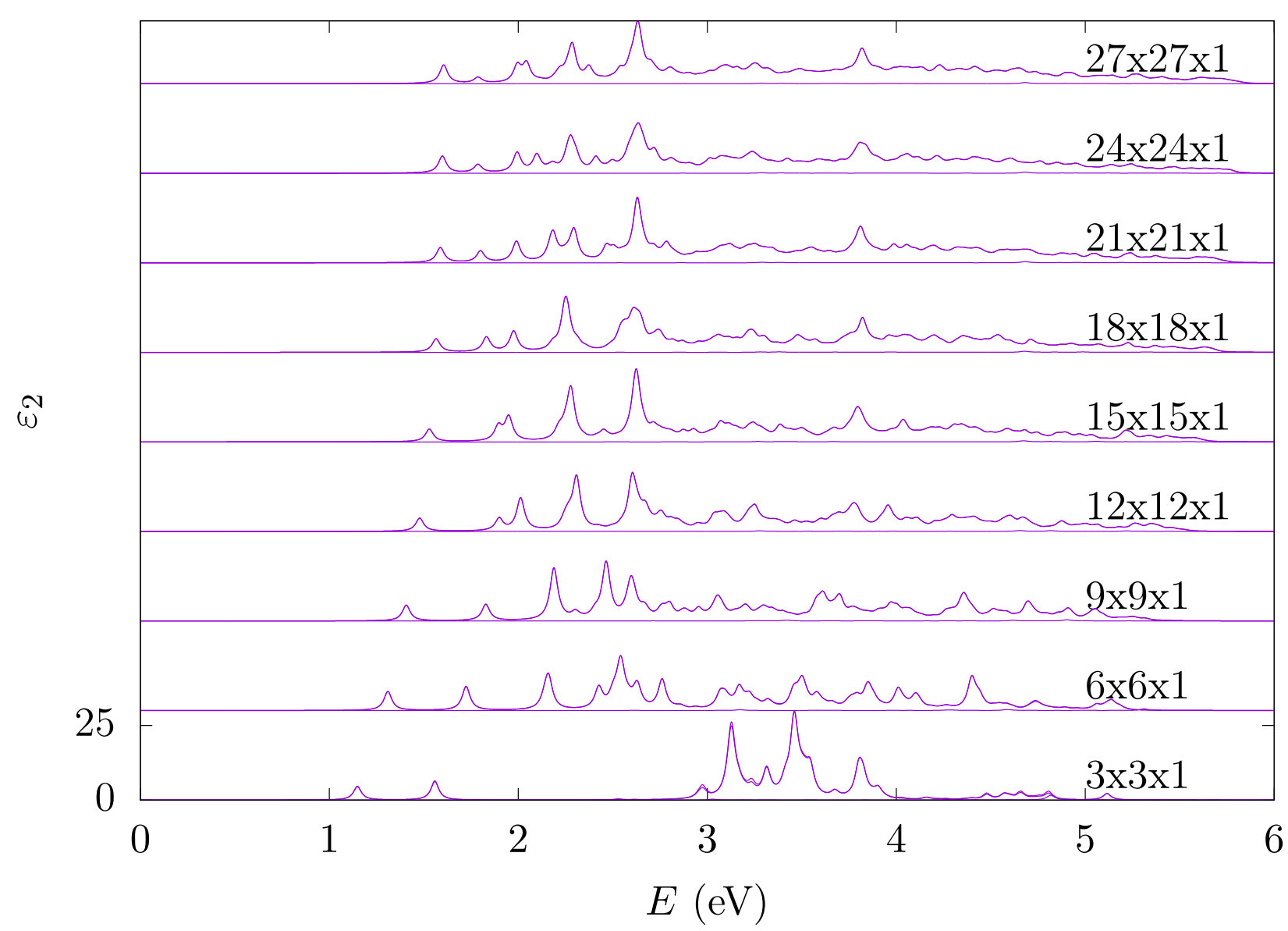

Figure S18: ε_2 of a $MoSe_2$ (top) and WSe_2 (bottom) monolayer as a function of the number of kpoints. $24 \times 24 \times 1$ kpoints for the monolayer correspond to $1 \times 24 \times 1$ kpoints for the lateral heterostructure with 12 f.u. $MoWSe_4$.

Table S1: Calculation parameters used for the GW+BSE calculations for the lateral heterostructure

parameter	value
ENCUTGW	30
KPOINTS	$24 \times 24 \times 1$ for the bilayer= $1 \times 24 \times 1$ for the LHS
NBANDS	192 for the bilayer (per f.u. $MoWSe_4$) = 2304 for the LHS
NBANDS BSE	20 VB, 20 CB
NOMEGA	30

References

(1) Sahoo, P. K.; Memaran, S.; Xin, Y.; Balicas, L.; Gutiérrez, H. R. One-pot growth of two-dimensional lateral heterostructures via sequential edge-epitaxy. *Nature* **2018**, *553*, 63–67.

(2) Kresse, G.; Furthmüller, J. Efficiency of ab-initio total energy calculations for metals and semiconductors using a plane-wave basis set. *Comput. Mater. Sci.* **1996**, *6*, 15–50.

(3) Perdew, J. P.; Burke, K.; Ernzerhof, M. Generalized Gradient Approximation Made Simple. *Phys. Rev. Lett.* **1996**, *77*, 3865–3868.

(4) Hedin, L. New method for calculating the one-particle Green's function with application to the electron-gas problem. *Phys. Rev.* **1965**, *139*, A796.

(5) Strinati, G. Application of the Green's Functions Method to the Study of the Optical Properties of Semiconductors. *Riv. Nuovo Cimento Soc. Ital. Fis.* **1988**, *11*, 1.

(6) Rohlfing, M.; Louie, S. G. Electron-hole excitations and optical spectra from first principles. *Phys. Rev. B* **2000**, *62*, 4927.

(7) Onida, G.; Reining, L.; Rubio, A. Electronic excitations: density-functional versus many-body Green's-function approaches. *Rev. Mod. Phys.* **2002**, *74*, 601.
